# Biogenesis and functions of aminocarboxypropyluridine in tRNA

**DOI:** 10.1038/s41467-019-13525-3

**Published:** 2019-12-05

**Authors:** Mayuko Takakura, Kensuke Ishiguro, Shinichiro Akichika, Kenjyo Miyauchi, Tsutomu Suzuki

**Affiliations:** Department of Chemistry and Biotechnology, Graduate School of Engineering, The University of Tokyo, 7-3-1 Hongo, Bunkyo-ku, Tokyo, 113-8656 Japan

**Keywords:** RNA modification, tRNAs

## Abstract

Transfer (t)RNAs contain a wide variety of post-transcriptional modifications, which play critical roles in tRNA stability and functions. 3-(3-amino-3-carboxypropyl)uridine (acp^3^U) is a highly conserved modification found in variable- and D-loops of tRNAs. Biogenesis and functions of acp^3^U have not been extensively investigated. Using a reverse-genetic approach supported by comparative genomics, we find here that the *Escherichia coli yfiP* gene, which we rename *tapT* (tRNA aminocarboxypropyltransferase), is responsible for acp^3^U formation in tRNA. Recombinant TapT synthesizes acp^3^U at position 47 of tRNAs in the presence of *S*-adenosylmethionine. Biochemical experiments reveal that acp^3^U47 confers thermal stability on tRNA. Curiously, the Δ*tapT* strain exhibits genome instability under continuous heat stress. We also find that the human homologs of *tapT*, *DTWD1* and *DTWD2*, are responsible for acp^3^U formation at positions 20 and 20a of tRNAs, respectively. Double knockout cells of *DTWD1* and *DTWD2* exhibit growth retardation, indicating that acp^3^U is physiologically important in mammals.

## Abstract (web summary)

*E. coli* and human tRNAs contain 3-(3-amino-3-carboxypropyl)uridine (acp^3^U) modification. Here the authors identify *E. coli* TapT and human DTWD1/2 as tRNA aminocarboxypropyltransferases responsible for acp^3^U formation. Inhibition of acp^3^U modification results in genome instability in heat-stressed *E. coli* and growth defects in human cells.

## Introduction

The emerging field of epitranscriptomics has revealed the chemical diversity and functional importance of RNA modifications. To date, about 150 species of RNA modifications have been identified in RNA molecules from all domains of life^1^. Transfer (t)RNAs are especially heavily modified; indeed, more than 80% of RNA modifications found so far were discovered in tRNA molecules from various organisms. By stabilizing tRNA tertiary structure and fine-tuning decoding capability, these modifications ensure that tRNAs function properly^2–4^.

A wide variety of tRNA modifications are found in the anticodon loop, especially at positions 34 and 37. These modifications stabilize and modulate codon–anticodon interactions on the ribosome, thereby ensuring accurate and efficient decoding during translation^2^. The second class of modifications is clustered in the tRNA core structure formed by the D-loop, TΨC-loop (T-loop), and variable-loop (V-loop). These tRNA modifications are structural modulators that contribute to correct folding and stabilization of tRNA^3^. Some of these modifications are also required for tRNA flexibility^5^. The 2′-*O*-methyl modifications found in the D- and T-loops, in particular, 2′-*O*-methylguanosine (Gm) at position 18, confer conformational rigidity on the tRNA core region by fixing C3′-*endo* ribose puckering^3,6^. Gm18 stabilizes the D-loop/T-loop interaction through base pairing with pseudouridine (Ψ) at position 55 in the T-loop^7,8^. Ψ55 stabilizes T-loop structure with additional hydrogen bond to the phosphate-ribose backbone^9^. 5-methyluridine (m^5^U, also known as ribothymidine) at position 54 in the T-loop confers thermal stability to tRNA^10^. In thermophilic organisms, additional modifications, including 5-methyl-2-thiouridine (m^5^s^2^U or s^2^T) and archaeosine (G+), are present in the tRNA core region^11–13^. These modifications stabilize tRNAs, enabling cell growth at high temperature^10,14,15^. The modifications in the core region play crucial roles in determining not only the physicochemical properties of tRNAs but also their cellular stability. Because properly modified mature tRNAs are required for accurate and efficient translation, living organisms have evolved a control quality system that degrades hypomodified tRNAs^16,17^.

**Fig. 1:**
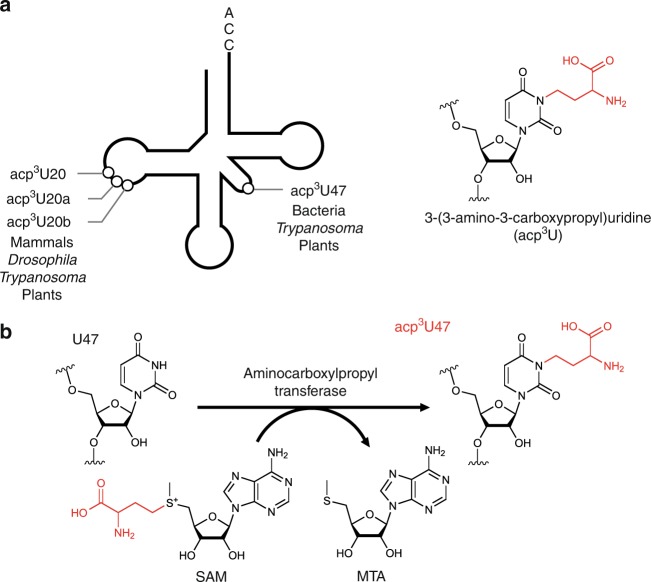
acp^3^U is a prevalent tRNA modification widely conserved in eukaryotes and bacteria. **a** Positions of acp^3^U modification in tRNA (left panel). Chemical structure of 3-(3-amino-3-carboxypropyl)uridine (acp^3^U) (right panel). The acp group is colored in red. acp^3^U is present at position 20 in mammals, *Drosophila* and *Trypanosoma*, at position 20a in mammals, *Drosophila* and plants, and at position 20b in plants. **b** Biosynthetic mechanism of acp^3^U modifications in tRNA and rRNA. The acp group of *S*-adenosylmethionine (SAM) is transferred to generate acp^3^U modification in RNA with release of *S*-methyl-5'-thioadenosine (MTA).

3-(3-amino-3-carboxypropyl)uridine (acp^3^U) is a widely conserved modification found in tRNA core region in bacteria and eukaryotes^1,18,19^ (Fig. 1a). The 3-amino-3-carboxypropyl (acp) group is attached to the N3 atom of the uracil base to prevent it from engaging in Watson–Crick base pairing. In *Escherichia coli*, acp^3^U is present at position 47 in the V-loop of tRNAs for Arg2, Ile1, Ile2, Ile2v, Lys, Met, Phe, Val2A, and Val2B (Fig. 1a)^20^. In eukaryotes, including human, rat and *Drosophila*, acp^3^U occurs at positions 20 and 20a in the D-loop of several cytoplasmic tRNAs (Fig. 1a)^20^. In *Trypanosoma brucei*, acp^3^U and its dihydrouridine derivative (acp^3^D) are present at positions 20 and 47, respectively, in cytoplasmic tRNA^Lys(TTT)^^21^(Fig. 1a). In several species of land plants, acp^3^U can be found at positions 20a and 20b in nuclear-encoded tRNAs, and at position 47 in tRNAs encoded in plastid and mitochondrial DNAs^20^.

An acp^3^U derivative, 1-methyl-3-(3-amino-3-carboxypropyl)pseudouridine (m^1^acp^3^Ψ), is present at the P-site of small subunit of the eukaryotic and archaeal ribosome: positions 1191 and 1248 in 18S rRNA from *S. cerevisiae* and human, respectively^22–24^. In human, m^1^acp^3^Ψ1248 is synthesized via three steps. The biogenesis is initiated by pseudouridylation mediated by H/ACA snoRNP bearing SNORA13^25^, followed by methylation catalyzed by EMG1^26^. Finally, TSR3 transfers the acp group of *S*-adenosylmethionine (SAM) to form m^1^acp^3^Ψ^27,28^. TSR3 contains a domain similar to SPOUT-class RNA methyltransferase^28^. The crystal structure of the archaeal homolog of TSR3 in complex with SAM revealed the molecular basis of acp^3^U formation^28^, although the enzymatic activity of TSR3 has never been described. Reduction in the level of the acp modification causes accumulation of 18S rRNA precursors and a reduction in the level of 40S ribosomal subunit, indicating that this modification is involved in 40S subunit assembly^28,29^.

Despite the high conservation of the acp^3^U modification in tRNAs from bacteria and eukaryotes, the biogenesis and physiological roles of this modification remain to be determined. The solution structure of the acp^3^U nucleoside does not affect its sugar conformation^30^. Structural analysis of D-acp^3^U-A trinucleotide suggested that acp^3^U20a binds to Mg^2+^, and this interaction may stabilize the local conformation of tRNA^31^. Chemical acetylation of the amino group of acp^3^U47 in *E. coli* tRNA^Phe^ does not affect its binding affinity for phenylalanyl-tRNA synthetase or the ribosome^32^. In the tertiary structure of tRNAs^33–35^, the acp group of acp^3^U47 is oriented toward the solvent side of the tRNA structure and does not directly interact with the other residues. In 1974, enzymatic formation of acp^3^U in tRNA was carried out using *E. coli* lysate^36^. Specifically, Nishimura’s group successfully reconstituted acp^3^U47 formation in tRNA^Phe^ in the presence of SAM, and demonstrated that the acp group of SAM is transferred to tRNA to form acp^3^U47 (Fig. 1b).

To achieve a deeper understanding of acp^3^U modification in tRNAs, it is necessary to identify the enzyme responsible for generating it. For systematic search for genes responsible for RNA modifications, we developed a genetic screening method called ribonucleome analysis, which takes advantage of mass spectrometric analysis of RNA modifications^37^. We use liquid chromatography/mass spectrometry (LC/MS) to systematically analyze total nucleosides of RNAs obtained from a series of strains harboring knockouts in uncharacterized genes. If a target RNA modification is absent in a certain knockout strain, we can identify the gene dedicated to biogenesis of the modification in a reverse-genetic manner. We have discovered dozens of genes responsible for RNA modifications in tRNAs^38–41^, rRNAs^42,43^, and mRNAs^44^. In this study, we apply ribonucleome analysis assisted by comparative genomics to identify an *E. coli* gene *yfiP* (renamed as *tapT*) responsible for acp^3^U47 formation. We successfully reconstitute formation of acp^3^U47 by recombinant TapT protein in the presence of SAM. Measurements of melting temperature reveal that acp^3^U47 stabilizes tRNA by 3 °C. We observe genome instability of the Δ*tapT* strain under continuous heat stress, indicating that this modification plays a physiological role in bacteria. We also identify eukaryotic homologs of *tapT*, including the human homologs *DTWD1* and *DTWD2*, and show that they are responsible for the formation of acp^3^U at positions 20 and 20a of tRNAs, respectively. In human cells, double knockout of *DTWD1* and *DTWD2* causes slow growth, indicating that acp^3^U is also physiologically important in mammals.

## Results

### *E. coli yfiP* is responsible for acp^3^U47 formation on tRNAs

**Fig. 2:**
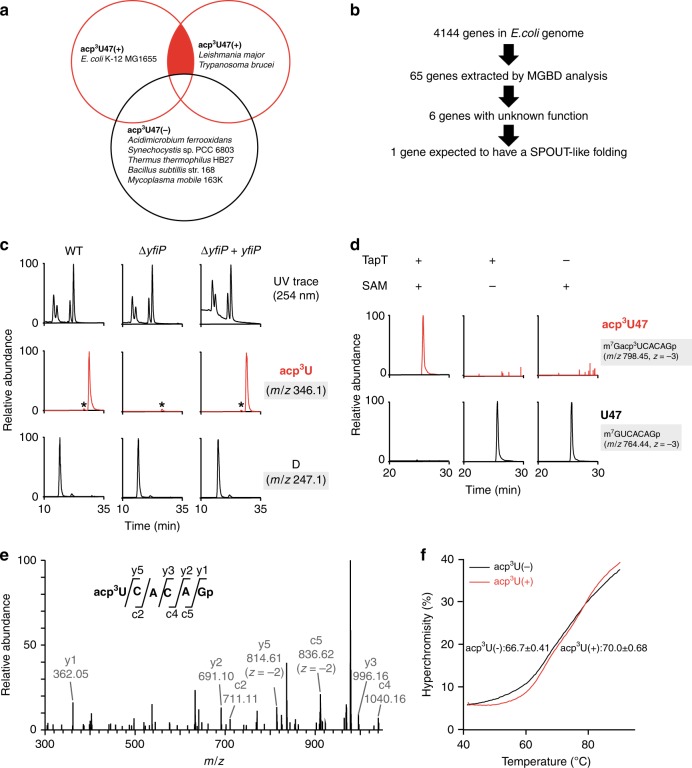
*yfiP* is responsible for acp^3^U47 formation in *E. coli*. **a** Venn diagram to narrow down the candidates by comparative genomics. Each circle represents the gene sets that are commonly present in the organisms with (+) or without (−) acp^3^U. Red circles represent groups of organisms that have acp^3^U47, whereas the black circle represents organisms that do not have acp^3^U47. Candidate genes lie in the red-filled region. **b** The process of narrowing down the candidate genes responsible for acp^3^U47 formation by comparative genomics. **c** Mass spectrometric nucleoside analyses of tRNA modifications from *E. coli* WT (left panels), Δ*yfiP* (center panels), an Δ*yfiP* rescued by plasmid-encoded *yfiP* (right panels). UV chromatogram (top) and extracted ion chromatograms (XICs) of the proton adducts of nucleosides for acp^3^U (middle) and D (bottom). Asterisks indicate nonspecific peaks with the same *m/z* value. **d** In vitro reconstitution of acp^3^U47 in *E. coli* tRNA^Met^ by recombinant TapT in the presence or absence of SAM. XICs show the indicated negative ions of the RNase T_1_-digested fragments with (top) or without (bottom) acp^3^U47. **e** Collision-induced dissociation spectrum of the acp^3^U-containing fragment of the tRNA^Met^ transcript, in which acp^3^U was reconstituted in vitro. The precursor ion is *m/z* 678.43. The product ions were assigned on the corresponding sequence as described^93^. The data confirmed the presence of acp^3^U at position 47. **f** Melting curves of the in vitro transcribed tRNA^Met^ with (red lines) or without (black lines) acp^3^U47 modification. *T*_m_ values were determined based on the inflection point of the melting curve. Data represent average values of triplicates, with s.d. *P* < 4.1 × 10^−3^ by Student’s *t* test. Source data are provided as a Source Data file.

To identify the gene responsible for acp^3^U47 formation in *E. coli*, we used comparative genomics to narrow down the candidate genes (Fig. 2a). Because little information was available about posttranscriptional modifications in the other bacterial tRNAs, we conducted nucleoside analyses of total RNAs from several bacterial species, *Acidimicrobium ferrooxidans*, *Synechocystis* sp. PCC 6803, *Thermus thermophilus* HB27, *Bacillus subtilis* str. 168, and *Mycoplasma mobile* 163K, and found that none of them contained acp^3^U (Supplementary Fig. 1). By contrast, in protists, acp^3^U is present in *T. brucei* cytoplasmic tRNA^Lys(UUU)^ ^21^, and we confirmed the presence of acp^3^U in the same tRNA from *Leishmania major*^45^. Using these data, we performed a comparative genomics analysis using the Microbial Genome Database^46^. Among 4144 *E. coli* ORFs, we selected 65 genes commonly present in *E. coli*, as well as in the two protists (*T. brucei* and *L. major*), but absent in the five bacteria mentioned above (Fig. 2a, b). Based on the annotation of each gene in the EcoCyc database^47^, we then narrowed down the list of candidates to six genes with unknown functions. Finally, we chose *yfiP* as a strong candidate for acp^3^U formation (Fig. 2b). *yfiP* belongs to unknown orthologous group COG3148 that contains an uncharacterized DTW domain. According to computational analyses, YfiP has a SPOUT-like methyltransferase structure^48^. Moreover, COG3148 was referred to the DTWD2 family (KOG4382), and classified into the TDD superfamily, which includes the TSR3 (COG2042), DTWD1 (KOG3795), and DTWD2 families^49^.

To examine whether *yfiP* is responsible for acp^3^U47 formation in tRNAs, we performed LC/MS nucleoside analysis of total RNAs from *E. coli* WT and Δ*yfiP* strains (Fig. 2c). As expected, no acp^3^U was detected in the Δ*yfiP* strain, but acp^3^U was restored by ectopic expression of a plasmid-encoded *yfiP* gene. These results clearly demonstrated that *yfiP* is essential for acp^3^U formation in *E. coli*. Hereafter, we refer to *yfiP* as *tapT* (tRNA aminocarboxypropyltransferase).

### TapT catalyzes acp^3^U47 formation

Next, we performed in vitro reconstitution of acp^3^U47 using the recombinant TapT protein. As a substrate, we isolated *E. coli* tRNA^Met^ lacking acp^3^U from the Δ*tapT* strain. After the reaction, the tRNA was digested by RNase T_1_ and analyzed by LC/MS. We clearly detected an RNA fragment containing acp^3^U in the presence of both TapT and SAM (Fig. 2d). TapT efficiently introduced acp^3^U47 on unmodified tRNA^Met^ transcribed in vitro (Supplementary Fig. 2), suggesting that the other tRNA modifications are not required for acp^3^U formation. The fragment was further probed by collision-induced dissociation, confirming that acp^3^U was introduced at position 47 (Fig. 2e). These data clearly demonstrated that TapT is a tRNA-modifying enzyme that catalyzes the SAM-dependent aminocarboxypropyl transfer reaction to form acp^3^U47.

### acp^3^U confers thermal stability on tRNA

Given that acp^3^U is a modification in the tRNA core region, we investigated whether acp^3^U47 stabilizes the tertiary structure of tRNA. We prepared *E. coli* tRNA^Met^ transcripts with or without acp^3^U47 and compared their melting profiles. As temperature increased, the tRNA gradually melted, and its hyperchromicity increased (Fig. 2f). The unmodified tRNA started melting around at 40 °C, whereas the tRNA containing acp^3^U47 remained stable even at 50 °C. The hyperchromicity of the modified tRNA was lower than that of the unmodified one throughout the course of the heating process. The melting temperature (*T*_m_) values of the tRNA with and without acp^3^U47 were 70.0 and 66.7 °C, respectively (Fig. 2f). Thus, a single acp^3^U modification thermally stabilized this tRNA by 3 °C.

### Motility defect in *E. coli* Δ*tapT* strain

**Fig. 3:**
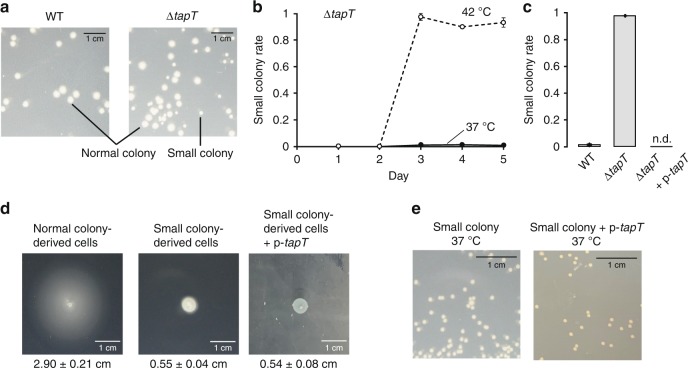
Lack of acp^3^U47 results in genome instability in *E. coli*. **a** Morphological alteration of WT (left) or Δ*tapT* (right) strains after cultivation at 42 °C for 4 days. Small colonies appeared in the Δ*tapT* strain. **b** Frequencies of small colony appearance of Δ*tapT* strain with continuous cultivation at 37 °C (closed circles) and 42 °C (open circles). Data represent average values of biological triplicates, with s.d. Source data are provided as a Source Data file. **c** Frequencies of small colonies in WT, Δ*tapT*, and Δ*tapT* overexpressing *tapT* after cultivation at 42 °C for 4 days. Data represent average values of biological triplicates, with s.d. Source data are provided as a Source Data file. **d** Motility test of the normal colony-derived cells (left), small colony-derived cells (middle), and small colony-derived cells overexpressing *tapT* (right). Diameter of halo was defined as the length of the line segment which passes through the center and the ends of the halo. Data represent average values of biological replicates (*n* = 5), with s.d. Source data are provided as a Source Data file. **e** Morphology of the small colony-derived cells (left) and the small colony-derived cells overexpressing *tapT* (right). Overnight saturated cultures were diluted, spotted onto LB plates, and cultivated at 37 °C.

To investigate the physiological roles of acp^3^U47 in *E. coli*, we explored the phenotypic features of the Δ*tapT* strain. Because acp^3^U47 thermally stabilizes tRNA, we compared the cell growth of WT and Δ*tapT* strains cultured at different temperatures (Supplementary Fig. 3a), but observed no significant difference in cell growth even at 42 °C. However, we happened to find a morphological phenotype unique to the Δ*tapT* strain when cultured continuously at high temperature. After 3 days cultivation at 42 °C, small colonies suddenly appeared in the Δ*tapT* strain (Fig. 3a, b), although no such colonies were detected in the first 2 days. Small colonies were not detected in the WT strain, in the Δ*tapT* strain cultured at 37 °C, or in the Δ*tapT* strain complemented with plasmid-encoded *tapT* gene (Fig. 3c).

We isolated and characterized small and normal-sized Δ*tapT* colonies cultured for 4 days at 42 °C, and then examined the growth and motility of these cells. We observed no significant difference in cell growth between small and normal colonies (Supplementary Fig. 3b). Cell motility was examined based on swimming activity on soft agar plates. As shown in Fig. 3d, cells derived from normal colonies swam well, and the diameter of the halo reached 2.90 ± 0.21 cm, whereas cells derived from small colonies did not move and instead remained near the original spot (0.55 ± 0.04 cm), suggesting that the small colony phenotype of the Δ*tapT* strain originates from a motility defect. When we sought to reverse the phenotype by introducing plasmid-encoded *tapT* into cells derived from small colonies, we observed no normal colonies on the plate (Fig. 3e). Supporting this result, lack of motility on soft agar was not restored by *tapT* complementation (0.54 ± 0.08 cm) (Fig. 3d). We confirmed restoration of acp^3^U in the small colony-derived Δ*tapT* strain by introduction of plasmid-encoded *tapT* gene (Supplementary Fig. 3c). Thus, the phenotype is caused by genomic mutations.

tRNA modification-deficient strains exhibit a moderate mutator phenotype mediated by the translational stress-induced mutagenesis (TSM) pathway^50,51^. Lack of *N*^6^-isopentenyladenosine (i^6^A) in tRNAs in the Δ*miaA* strain induces transversion-type DNA mutation^50^. If similar translation stress is induced by loss of acp^3^U in the Δ*tapT* strain, the genomic mutation linked to the small colony phenotype might be enhanced by the TSM pathway^51^. To measure the genomic mutation rate of the Δ*tapT* strain, we performed a mutator assay^52^. Specifically, we cultured the cells continuously at 42 °C and then examined their resistance to nalidixic acid (Supplementary Fig. 4). As a positive control, we used the Δ*mutS* strain, in which the DNA mismatch repair system is inactive;^53^ a number of small colonies arose on the plate, whereas the negative control strain (Δ*yagA*) showed a lower mutation rate. As reported, the Δ*miaA* strain exhibited a mild mutator phenotype. However, in contrast to our speculation, the mutation rate of the Δ*tapT* strain was in the same range as that of the control strain, suggesting that the small colony phenotype of the Δ*tapT* strain under heat stress cannot be explained by a mutator phenotype.

### DTWD1 and DTWD2 mediate acp^3^U formation in human tRNAs

**Fig. 4:**
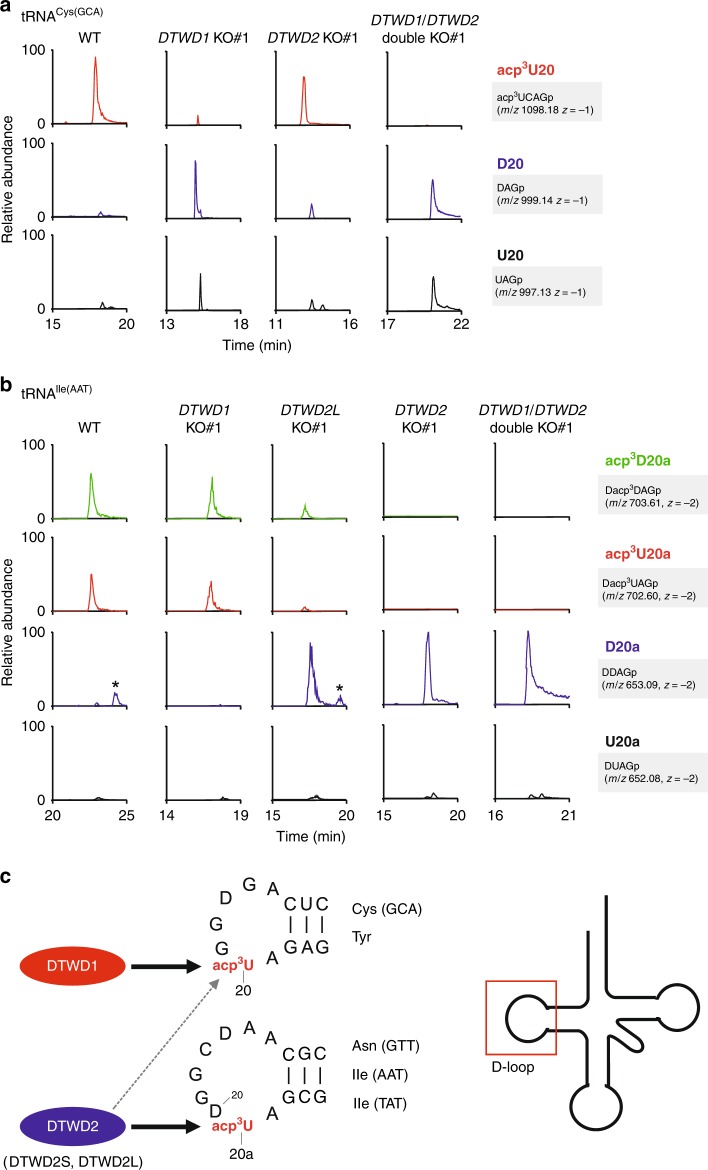
Human *DTWD1* and *DTWD2* are responsible for acp^3^U formation. **a** Mass spectrometric analyses of acp^3^U20 in tRNA^Cys(GCA)^ isolated from WT (left), *DTWD1* KO (left middle), *DTWD2* KO (right middle), and *DTWD1*/*DTWD2* double-KO (right) strains. XICs show corresponding negative ions of the RNase T_1_-digested fragments as indicated on the right-hand side of each chromatogram. Mass spectra of acp^3^D- or D-containing fragments overlap with the isotopic peaks of acp^3^U- or U-containing fragments. The peak intensities of these fragments are normalized in consideration of those isotopes. Asterisk indicates nonspecific peaks with the same *m/z* value. **b** Mass spectrometric analyses of acp^3^U20a in tRNA^Ile(AAT)^ isolated from WT (left), *DTWD1* KO (left middle), *DTWD2L* KO (middle), *DTWD2* KO (right middle), and *DTWD1*/*DTWD2* double-KO (right) strains. **c** Substrate specificity of human DTWD1 and DTWD2. DTWD1 is responsible for acp^3^U20 formation in tRNAs for Cys(GCA) and Tyr, whereas DTWD2 is responsible for acp^3^U20a formation in tRNAs for Asn(GTT), Ile(AAT), and Ile(TAT). DTWD2 also has a weak activity to form acp^3^U20. The sequence of tRNA^Cys^ and tRNA^Asn^ are shown.

In mammals, acp^3^U is present at position 20 of tRNA^Tyr^ ^54^ and position 20a of tRNA^Asn^ ^55^. We reanalyzed high throughput sequencing of human tRNAs^56^ and observed an apparent U-to-C conversion at positions 20 and 20a of these tRNAs, indicating that acp^3^U causes misincorporation during cDNA synthesis. Based on the misincorporation signatures at positions 20 and 20a of several human tRNAs, we predicted the presence of acp^3^U20 in two tRNAs for Cys(GCA) and Tyr, and acp^3^U20a in three tRNAs for Asn(GTT), Ile(AAT), and Ile(TAT). We then isolated these cytoplasmic tRNAs from HEK293T cells and analyzed the tRNA modifications by capillary LC–nanoelectrospray ionization (ESI)–MS (RNA-MS)^37^. We confirmed the presence of acp^3^U at positions 20 and 20a in these five tRNA species (Fig. 4a, b and Supplementary Fig. 5a–c). In addition, we found acp^3^D at position 20a of tRNA^Ile(AAT)^ (Fig. 4b).

As described above, *tapT* belongs to the *DTWD2* family and TDD superfamily^49^. Two paralogs of DTW-containing proteins in the TDD superfamily, DTWD1 and DTWD2, are encoded in the human genome, and we hypothesized that these proteins are responsible for acp^3^U at positions 20 and 20a. To examine this speculation, we knocked out each of these proteins in HEK293T cells using the CRISPR-Cas9 system. Two isoforms of human *DTWD2* with different first exons are produced by alternative splicing: *DTWD2L* (long) and *DTWD2S* (short). (Supplementary Fig. 6a). To delete both isoforms, we designed sgRNAs targeting exons 2 and 3 (Supplementary Fig. 6a). We also constructed *DTWD2L*-specific KO cell line by targeting the exon 1 of *DTWD2L*. Finally, we constructed *DTWD1*/*DTWD2* double-KO cell lines. Genotyping of each KO cell line confirmed frameshift mutations in both alleles (Supplementary Fig. 6b).

We then isolated five individual tRNAs bearing acp^3^U from a series of KO strains and examined their modification status by RNA-MS. In the *DTWD1* KO cells, the level of acp^3^U20 in two tRNAs for Cys(GCA) and Tyr were drastically reduced, with the modified bases converted to D20 and U20, although a small percentage of acp^3^U20 remained (Fig. 4a and Supplementary Fig. 5a). We observed no effect on acp^3^U20a or acp^3^D20a in the other three tRNAs. No acp^3^U20 was detected in the *DTWD1*/*DTWD2* double-KO cells (Fig. 4a and Supplementary Fig. 5a), indicating that DTWD1 is primarily responsible for introducing acp^3^U20, whereas DTWD2 plays a supportive role in acp^3^U20 formation (Fig. 4c). On the other hand, acp^3^U20a and acp^3^D20a were deficient, and replaced by D20a, in three tRNAs for Asn(GTT), Ile(AAT), and Ile(TAT) in the *DTWD2* KO cells (Fig. 4b and Supplementary Fig. 5b, c), suggesting that DTWD2 has a strong specificity for introducing acp^3^U20a and acp^3^D20a (Fig. 4c). These data demonstrated that DTWD1 and DTWD2 have different substrate preferences for tRNAs, and are responsible for the biogenesis of acp^3^U20 and acp^3^U20a, respectively.

Regarding two isoforms of *DTWD2*, we isolated tRNA^Ile(AAT)^ from *DTWD2L* KO cells, and analyzed the status of acp^3^U20a modification (Fig. 4b). Both acp^3^U20a and acp^3^D20a partially decreased, and instead D20a increased. These data clearly demonstrated that DTWD2L actually has an activity for acp^3^U(D)20a formation, and DTWD2S is redundantly responsible for the remaining modification.

Intriguingly, D20 or D20a appeared upon knockout of *DTWD1* or *DTWD2* (Fig. 4a, b and Supplementary Fig. 5a–c). D20 and D20a are present in other tRNA species that do not contain acp^3^U and have been proposed to be introduced by DUS2 and DUS4L, respectively^57,58^. Thus, DTWD1/2-mediated acp^3^U formation inhibits D20(a) formation.

### Growth reduction of *DTWD1* and *DTWD2* double knockout cells

**Fig. 5:**
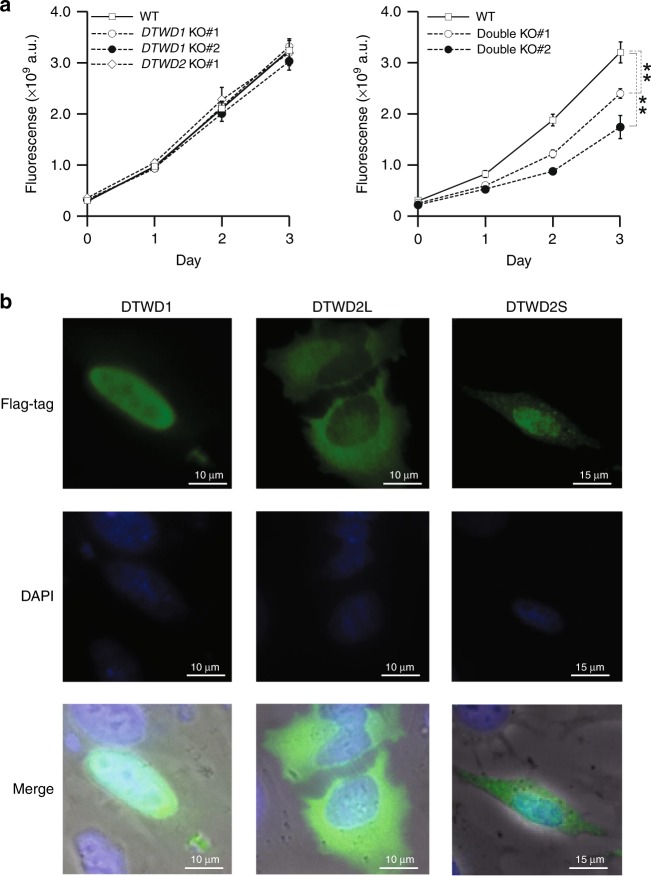
Physiological role and subcellular localization of DTWD1 and DTWD2. **a** Growth curves of WT HEK293T (open square), *DTWD1* KO#1 (open circle), *DTWD1* KO#2 (closed circle) and *DTWD2* KO (open rhombus) strains (left panel), and WT HEK293T (open square), *DTWD1*/*DTWD2* double-KO#1 (open circle) and *DTWD1*/*DTWD2* double-KO#2 (closed circle) strains (right panel), cultured in DMEM medium. Fluorescence was calculated as the average values of biological replicates (*n* = 6), with s.d. **P < 8.1 × 10^−6^ by Student’s *t* test. Source data are provided as a Source Data file. **b** Subcellular localization of DTWD1 (left panels), DTWD2L (middle panels), and DTWD2S (right panels) in HeLa cells immunostained with an anti-FLAG antibody (green). Nuclei were stained with DAPI (blue). Images were superimposed to generate the merged panels.

We next investigated the physiological significance of acp^3^U modifications in human tRNAs. In comparison with WT HEK293T cells, little growth reduction was observed in *DTWD1* and *DTWD2* single-KO cells (Fig. 5a), whereas the *DTWD1*/*DTWD2* double-KO cells exhibited a severe growth phenotype (Fig. 5a), indicating that *DTWD1* and *DTWD2* play redundant roles in supporting normal cell growth. Given that tRNA modifications are required for the cellular stability of tRNAs^16^, we asked whether loss of acp^3^U modification affects the steady-state level of tRNAs. To this end, we performed Northern blotting to compare the steady-state levels of four tRNAs between WT HEK293T and *DTWD1*/*DTWD2* double-KO cells (Supplementary Fig. 7a, b). No significant difference was observed for any tRNAs, suggesting that loss of acp^3^U does not affect tRNA stability.

Finally, we analyzed the subcellular localization of DTWD1 and DTWD2. For this purpose, FLAG-tagged DTWD1, DTWD2L, and DTWD2S were transiently expressed in HeLa cells and detected by immunostaining (Fig. 5b). DTWD1 was predominantly localized in nucleus, suggesting that DTWD1-mediated acp^3^U20 formation takes place in nucleus. By contrast, the two isoforms of DTWD2 exhibited different subcellular localization: DTWD2L was cytoplasm, whereas DTWD2S was widely diffused throughout the cells but concentrated in the nucleus. Thus, acp^3^U20a formation occurs in both the cytoplasm and the nucleus.

## Discussion

In this study, we discovered that the *tapT* gene is responsible for acp^3^U47 formation in *E. coli* tRNAs. TapT was the last unknown tRNA-modifying enzyme in *E. coli*. We successfully reconstituted in vitro acp^3^U47 formation with the recombinant TapT protein in the presence of SAM, revealing that TapT catalyzes acp transfer from SAM to tRNA. The acp moiety of SAM is also utilized for the synthesis of various biomolecules^59^, including bacterial betaine lipid, nocardicin, microcin C (McC^1177^), and diphthamide formed in EF-2. In other tRNA modification, the acp transfer reaction is involved in the synthesis of wybutosine (yW), a hypermodified nucleoside present in eukaryotic tRNA^Phe^^16^. The SAM-dependent class I methyltransferase TYW2 catalyzes the acp transfer reaction and synthesizes yW-86, a precursor of yW^40,60^. However, TDD superfamily proteins do not have sequence homology with these acp-transferases^49^. According to the crystal structure of archaeal Tsr3, which is a member of TDD superfamily protein^28^, it has a SPOUT-class RNA methyltransferase fold. The closest structural homolog of the TDD superfamily is a tRNA methyltransferase Trm10. Further structural studies of TapT and DTWD1/2 are necessary to reveal the molecular basis of tRNA substrate recognition and acp^3^U formation.

Measurement of melting temperature revealed that a single acp^3^U47 conferred thermal stability to tRNA, increasing the *T*_m_ value by 3 °C. In the tertiary structures of tRNAs^33–35^, the acp group of the modification does not interact with any other residues but resides in close vicinity to the backbone of the T-stem. We speculated that the acp group might stabilize the local conformation of tRNA by interacting with the T-stem via magnesium ion or water^31^. In addition, the acp group at the N3 atom position hinders Watson–Crick base pairing and may destabilize some metastable structures so as to ensure correct folding of tRNA, similarly to the function of m^1^A9^61^.

Strikingly, the *E. coli* Δ*tapT* strain exhibited a small colony phenotype associated with motility defect under continuous heat stress. Because ectopic expression of *tapT* did not restore the phenotype, we speculated that genomic mutations in swarming-related genes might be involved. Curiously, translation stress in *E. coli* strains bearing mutations in tRNA or the tRNA-modifying enzyme causes mutator phenotypes^62,63^. These facts prompted us to speculate that loss of acp^3^U in the Δ*tapT* strain contributes to the small colony phenotype mediated by the TSM pathway^51^. Unexpectedly, however, Δ*tapT* strain did not exhibit any mutator phenotypes under continuous heat stress conditions, indicating that neither error-prone replication nor aberrant DNA repairing is involved in this phenotype. Dysregulation of transposable elements are another possible source of genome instability^64^, but this should be investigated in future studies.

To investigate the phylogenetic distribution of the TDD family, we generated a phylogenetic tree of organisms possessing or lacking each homolog (Supplementary Fig. 8a). TSR3 orthologs predominated in eukaryotes and archaea, not in bacteria. Among the eukaryotes, DTWD1 orthologs are only present in vertebrates, insects, nematodes, and protists, but not in fungi and plants. DTWD2 orthologs are divided into two subfamilies, eukaryotic and bacterial (TapT). Eukaryotic DTWD2 orthologs are present in vertebrates, protists, and plants, whereas bacterial DTWD2 (TapT) orthologs are present in Bacteroidetes, Proteobacteria, Actinobacteria, Cyanobacteria, and Firmicutes, as well as in vertebrates, plants, and protists. We constructed multiple alignment of DTWD1 and DTWD2 homologs from representative organisms, and draw a phylogenetic tree using the maximum likelihood method (Supplementary Fig. 8b). DTWD1 orthologs form a small subfamily, clearly separated from the larger DTWD2 family with 100% bootstrap probability. This tree clearly reveals two major subfamilies of DTWD2: eukaryotic DTWD2, which is responsible for acp^3^U formation in the D-loop; and bacterial DTWD2 (TapT), which is responsible for acp^3^U formation in the V-loop.

*T. brucei* tRNA^Lys^ contains acp^3^U20 and acp^3^D47^21^. Consistent with this finding, one DTWD1 ortholog (Tb09.244.2810) and one DTWD2 ortholog (Tb927.3.4690) are encoded in the *T. brucei* genome^49^, indicating that these proteins are responsible for forming acp^3^U20 and acp^3^D47, respectively.

Recently, comprehensive analyses of *Vibrio cholerae* tRNAs identified acp^3^U and its derivative at positions 20b, 46, and 47^65^. Curiously, the *Vibrio cholerae* genome encodes three DTWD2 paralogs (VC1533, VC0131, and VC1980). According to the phylogenetic analysis (Supplementary Fig. 8b), VC1533 is a bacterial DTWD2 (TapT) ortholog that is assumed to be responsible for acp^3^U47 formation. Given that VC0131 branches from the root of the bacterial DTWD2 (TapT) subfamily, this homolog might be responsible for acp^3^U46 formation. VC1980 forms a unique subfamily with other bacterial orthologs near the eukaryotic DTWD2 subfamily. Considering that eukaryotic DTWD2 is responsible for acp^3^U20a formation, we speculated that the VC1980 subfamily is responsible for acp^3^U20b formation. *Pseudomonas* species contain two DTWD2 paralogs (Supplementary Fig. 8b). *P. aeruginosa* PA3606 and PA1424 belong to the bacterial TapT and VC1980-like subfamilies, respectively, and might be responsible for acp^3^U47 and acp^3^U20b formation.

In plants, acp^3^U20a and acp^3^U20b are found in nuclear-encoded tRNAs, while acp^3^U47 is found in tRNAs encoded in plastid and mitochondrial DNAs^20^. *Arabidopsis thaliana* has three paralogs of DTWD2 (Supplementary Fig. 8b). AT2G41750 is a typical eukaryotic DTWD2 responsible for acp^3^U20a formation. AT1G03687, along with other plant orthologs, forms a subfamily closely related to the bacterial DTWD2 (TapT) subfamily, and this paralog is predicted to localize in chloroplasts, implying that it is responsible for acp^3^U47 formation in plastid tRNAs. AT5G54880, along with other plant orthologs, forms a subfamily near the VC1980-like subfamily, implying that this paralog is required for acp^3^U20b formation. Experimental verification is necessary to confirm these speculations.

Double knockout of *DTWD1* and *DTWD2* caused severe growth defects in culture cells, indicating the physiological importance of acp^3^U modification. Although the functional roles of this modification remain elusive, it has been suggested that acp^3^U20a can coordinate Mg^2+^, thereby stabilizing the local conformation of tRNA^31^. The stabilization of tRNAs by acp^3^U20/20a may lead to efficient translation and rapid growth. According to the Genotype-Tissue Expression project (Supplementary Fig. 9a, b)^66^, *DTWD1* and *DTWD2* are differentially expressed in various tissues, indicating that the modification frequencies of acp^3^U20/20a might vary. Intriguingly, the two isoforms of DTWD2 exhibited different subcellular localization: DTWD2L and DTWD2S predominantly localized in the cytoplasm and nucleus, respectively. The expression levels of these two isoforms also varied among tissues (Supplementary Fig. 9b). Although expression of DTWD2L is low in many tissues, it is upregulated in others, including testis (Supplementary Fig. 9b). Because the different subcellular localizations of the two isoforms might affect tRNA maturation, tissue-specific isoform expression implies that acp^3^U modification is biologically important.

Loss of tRNA modification results in pathological consequences. We previously reported that human mitochondrial diseases are caused by hypomodification of mitochondrial tRNAs^67,68^ and proposed “RNA modopathy” as a distinct category of human diseases^69^. Recent whole-exome sequencing analyses identified loss-of-function mutations in many genes responsible for tRNA modifications from patients with a wide range of diseases, including neurological disorders, cancers, and diabetes^58,70–73^. *DTWD1* is a target of p53 and works as a tumor suppressor gene for gastric cancer^74^ and is downregulated in some cancer cell line. HDAC3 regulates p53-mediated expression of *DTWD1*. In primary melanomas, high expression of *DTWD1* mediated by HIF-1α correlates with poor prognosis and shorter disease-free status^75^. Mutation in *DTWD1* is also a risk factor for bipolar disease^76^. Copy number variation in the genome region containing *DTWD2* has been observed in primary open-angle glaucoma^77^. Further studies will be necessary to understand the molecular pathogenesis of human diseases associated with loss of acp^3^U modification in tRNAs.

## Methods

### Bacterial strains, media, and plasmid construction

Δ*yfiP* (JW5409) or Δ*yagA* (JW0260) with kanamycin resistance markers (Keio collection)^78^ and their parental *E. coli* strain BW25113 (obtained from the Genetic Stock Research Center, National Institute of Genetics, Japan) were cultivated in LB medium with vigorous shaking. *A. ferrooxidans* (obtained from the Biological Resource Center, National Institute of Technology and Evolution, Japan) was cultured in 0.5 g/L MgSO_4_·7H_2_O, 0.4 g/L (NH_4_)_2_SO_4_, 0.2 g/L K_2_HPO_4_, 0.1 g/L KCl, 10 mg/L FeSO_4_·7H_2_O, and 0.025% yeast extract (adjusted to pH 2.0 with 2 M H_2_SO_4_) at 45 °C^79^. *Synechocystis* sp. PCC 6803 (kindly provided by K. Sonoike of Waseda University, Japan) was cultured with BG11 medium^80^ under LED light at 34 °C. *T. thermophilus* HB27 (kindly provided by N. Shigi of AIST, Japan) was cultured in rich medium containing 0.8% polypeptone, 0.4% yeast extract, and 0.3% NaCl (adjusted to pH 7.5 with 1 M HCl) at 70 °C rich medium as previously described^81^. *B. subtilis* str.168 (kindly provided by A. Soma of Chiba University, Japan) was cultured in LB medium at 37 °C^82^. *M. mobile* 163K (kindly provided by M. Miyata of Osaka City University, Japan) was grown in Aluotto medium (pH 7.8), consisting of 2.1% heart infusion broth (Difco), 0.56% yeast extract, 10% horse serum (inactivated at 56 °C), and 0.005% ampicillin at 25 °C^83^.

To construct a plasmid encoding *yfiP* gene for complementation, the open reading frame of *yfiP* with promoter region was PCR-amplified from the *E. coli* genome using a set of primers listed in Supplementary Table 1. Amplified products were inserted into the HindIII/EcoRI sites of the pMW118 vector (Invitrogen).

### Human cell culture and measurement of cell proliferation

HEK293T (CRL-11268, ATCC) and Hela (CCL-2, ATCC) cells were cultured in high-glucose DMEM (D5796; Sigma) supplemented with 5% FBS (Gibco) and 1% penicillin–streptomycin (Fujifilm Wako Pure Chemical Corporation), at 37 °C in a humidified atmosphere containing 5% CO_2_. To compare cell proliferation, WT HEK293T cells and a series of knockout cells were seeded into 96-well plates (4.0 × 10^3^ cells per well). Each day, a 1/10th volume of 1 mM resazurin solution in PBS was added to each well, and the cells were incubated for 3 h. The fluorescence of reduced resazurin was measured using a fluorescence microplate reader (SpectraMax Paradigm MultiMode Detection Platform; Molecular Devices) with excitation/emission at 560/590 nm.

### Construction of human *DTWD1/DTWD2* KO cell lines

*DTWD1* and *DTWD2* single- and double-KO cells were generated using the CRISPR-Cas9 system, as described^69,84^. Two sgRNAs targeting exon 1 of the *DTWD1* gene, and two sgRNAs targeting exon 2 or 3 of the *DTWD2* gene, were designed. Sense and antisense oligonucleotides for the sgRNAs (Supplementary Table 1) were cloned into the pX330 vector (Addgene plasmid 42230)^85^. HEK293T cells (1.0 × 10^5^ cells per plate) were transfected with the vector bearing the sgRNA sequence, along with pEGFP-N1 (Clontech) and modified pLL3.7 vectors harboring a puromycin resistance gene. One day after transfection, the cells were seeded at low density and selected with 1 μg/mL puromycin. The KO cell lines were selected by confirming the frameshift mutations in the target region. *DTWD1*/*DTWD2* double-KO cells were generated from the *DTWD1* single-KO cells.

### RNA extraction and isolation of individual tRNAs

Total RNAs of *E. coli* were extracted as described^86^. Total RNAs of *A. ferrooxidans*, *Synechocystis* sp. PCC 6803, *T. thermophilus* HB27, *B. subtilis* str.168, and *M. mobile* 163K were extracted using the acidic phenol method as described^79,83^. Total RNAs were extracted from HEK293T cells using TriPure Isolation Reagent (Roche Life Science).

Individual tRNAs were isolated by reciprocal circulating chromatography as described^87^ using the 5′-terminal ethylcarbamate amino-modified DNA probes (Sigma Aldrich Japan) listed in Supplementary Table 1.

### Mass spectrometric analysis

For nucleoside analysis of *E. coli*, total RNAs (1.6 μg) were digested with 0.8 U nuclease P1 (FUJIFILM Wako Pure Chemical) at 37 °C for 60 min. The digests were adjusted to 50 mM ammonium bicarbonate (pH 8.2), followed by addition of 0.2 U phosphodiesterase I (Worthington) and incubated at 37 °C for 60 min, then 0.2 U of bacterial alkaline phosphatase (BAP, from *E. coli* C75, FUJIFILM Wako Pure Chemical) was added, and incubated at 37 °C for 60 min, as described^86^. Nucleosides were dissolved in 90% acetonitrile (10% water), and subjected to a hydrophilic interaction LC (ZIC-cHILIC, 3 μm particle size, 2.1 × 150 mm, Merck) coupled with ESI-MS on a Q Exactive hybrid Quadrupole-Orbitrap mass spectrometer (Thermo Fisher Scientific), equipped with an ESI source and an Ultimate 3000 liquid chromatography system (Thermo Fisher Scientific)^88^. The mobile phase consisted of 5 mM ammonium acetate (pH 5.3) (solvent A) and acetonitrile (solvent B), and the nucleosides were chromatographed with a flow rate of 100 μL/min in a multistep linear gradient; 90–40% B from 0 to 30 min, 40% B for 10 min, and then initialized to 90% B. Proton adducts of nucleosides were scanned in a positive polarity mode over an *m/z* range of 103–700. Xcalibur 3.0.63 (Thermo Fisher Scientific) was used for the system operation.

Nucleoside analyses for comparative genomics were performed by reverse-phase chromatography/ESI-MS. Total RNAs (16–160 µg) were digested in a reaction mixture containing with 20 mM ammonium acetate (pH 5.3), 0.04–0.08 U BAP, and 0.05–0.1 U nuclease P1, and subjected to InertSil ODS-3 (5 μm particle size, 2.1 × 250 mm, GL sciences) or SunShell C18 (2.6 μm particle size, 2.1 × 150 mm, ChromaNik Technologies) column, then analyzed by an LCQ Advantage ion-trap mass spectrometer (Thermo Fisher Scientific) with an ESI source and an HP1100 liquid chromatography system (Agilent Technologies)^79^ or a Q Exactive hybrid Quadrupole-Orbitrap mass spectrometer (Thermo Fisher Scientific) equipped with an ESI source and an Ultimate 3000 liquid chromatography system (Thermo Fisher Scientific)^88^. The mobile phase consisted of 5 mM ammonium acetate (pH 5.3) (solvent A) and acetonitrile (solvent B). Xcalibur 2.0 SUR1 (Thermo Fisher Scientific) was used for operation of the LCQ Advantage system,.

For RNA fragment analysis, the isolated tRNAs (2 pmol) were digested with 10 U of RNase T_1_ in 20 mM ammonium acetate (pH 5.3) at 37 °C for 60 min. The digests were mixed with 1/10 vol. of 0.1 M triethylamine acetate (pH 7.0) and subjected to LC/nano ESI-MS on an LTQ Orbitrap mass spectrometer (Thermo Fisher Scientific) equipped with a splitless nanoflow high-performance LC (nano-HPLC) system (DiNa, KYA Technologies) using a nano-LC trap column (C18, 0.1 × 0.5 mm, KYA Technologies) and a capillary column (HiQ Sil C18W-3, 0.1 × 100 mm, KYA Technologies) as described^37^. Digested fragments were separated for 40 min at a flow rate of 300 nL/min by capillary LC using a linear gradient from 2 to 100% solvent B (v/v) in a solvent system consisting of 0.4 M 1,1,1,3,3,3-hexafluoro-2-propanol (HFIP) (pH 7.0) (solvent A) and 0.4 M HFIP (pH 7.0) in 50% methanol (solvent B). The eluent was ionized by ESI source in a negative polarity and scanned over an *m/z* range of 600–2000. Xcalibur 2.0.7 (Thermo Fisher Scientific) was used for the system operation.

The LC/MS data were analyzed using Xcalibur Qual browser (Thermo Fisher Scientific). In nucleoside analysis, the peak of modified nucleoside was normalized by the peak area of the unmodified nucleoside. In RNA fragment analysis, the peak of each fragment was normalized by the sum of modified and unmodified fragments. Mongo Oligo Mass Calculator v2.08 (https://mods.rna.albany.edu/masspec/Mongo-Oligo) was used for assignment of the product ions in CID spectra.

### Expression and purification of recombinant protein

To construct an expression plasmid for TapT recombinant protein, the open reading frame of *yfiP* was PCR-amplified from the *E. coli* genome using the primers listed in Supplementary Table 1. The amplified products were inserted into the NdeI/XhoI sites of the pET28a vector (Invitrogen) to yield pET-TapT, which expresses the N-terminally His-tagged TapT protein. The *E. coli* BL21(DE3) strain was transformed with the plasmid and cultured at 37 °C. When the absorbance at 600 nm (*A*_600_) reached 0.5, isopropyl-β-D-thiogalactopyranoside was added to a final concentration of 0.1 mM for inducing expression, and the cells were cultured at 18 °C for an additional 24 h. The cells were harvested and lysed by sonication in lysis buffer [20 mM HEPES-KOH (pH 7.5), 500 mM KCl, 6 mM β-mercaptoethanol (β-Me), and 1 mM phenylmethanesulfonyl fluoride]. The soluble recombinant protein was purified using a HisTrap HP column (GE Healthcare) and anion exchange chromatography (Mono-Q HR 5/5, GE Healthcare) using the AKTA Purifier 10 system (GE Healthcare). The purified protein was dialyzed against a buffer consisting of 20 mM HEPES-KOH (pH 7.5), 200 mM KCl, 6 mM β-Me, and 10% glycerol, and then stored at −80 °C.

### In vitro reconstitution of acp^3^U by TapT

For the substrates, we isolated tRNA^Met^ from the Δ*tapT* strain by reciprocal circulating chromatography^87^. The tRNA^Met^ transcript was prepared by in vitro transcription using T7 RNA polymerase, essentially as described^89^. The synthetic DNAs used for template construction by PCR are listed in Supplementary Table 1. In vitro transcription was performed at 37 °C overnight in a reaction mixture containing 40 mM Tris-HCl (pH 7.5), 24 mM MgCl_2_, 5 mM DTT, 2.5 mM spermidine, 0.01% Triton X-100, 30 nM DNA template, 2 mM GTP, 2 mM CTP, 2 mM UTP, 2 mM ATP, and 10 mM GMP. Subsequently, the reaction mixture was subjected to phenol–chloroform extraction and desalted by passing through a PD-10 columns (GE Healthcare). The transcribed tRNA was purified by running on 10% denaturing polyacrylamide gel electrophoresis (PAGE).

In vitro reconstitution of acp^3^U modification was performed at 37 °C for 2 h in a reaction mixture containing 20 mM HEPES-KOH (pH 7.5), 10 mM MgCl_2_, 100 mM NaCl, 1 mM DTT, 1 mM SAM, 8.5 μM tRNA^Met^, and 0.75 μM recombinant TapT. The tRNA product was digested with RNase T_1_ (Thermo Fisher Scientific) and subjected to LC/MS analysis as described above.

### Measurement of thermal stability of tRNAs

The tRNA (2.5 μg) was dissolved in degassed buffer containing 20 mM HEPES-KOH (pH 7.5), 150 mM NaCl, and 6 mM MgCl_2_, and incubated at 75 °C for 5 min, followed by cooling at room temperature for annealing. A UV–Vis spectrophotometer (V-630; JASCO) equipped with an 8 multi-quartz micro cell array (path length: 10 mm; JASCO) was used to monitor the hyperchromicity of tRNA. The temperature gradient was as follows: 25 °C for 30 s, and then ramped at 5 °C/min to 40 °C and held for 5 min, and ramped at 0.5 °C/min to 90 °C.

### Morphological analysis under heat-stressed conditions

*E. coli* strains were cultivated in 5 mL LB medium with vigorous shaking at 37 °C or 42 °C for 16–24 h until reaching the late stationary phase. Then, cells were diluted by a factor of 10^6^ or 10^7^ so that the concentration was suitable for counting the colony numbers. One hundred microliters of the diluted cells were spread on LB agar plate and cultured at 37 °C overnight. In parallel, 2.5 μL harvested cells was inoculated into 5 mL fresh LB medium and cultured at the same temperature. The process described above was repeated for 5–7 days. The frequency of small colonies appearing on the plate was calculated by counting the number of normal and small colonies on each plate.

### Motility assay

The motility assay was performed basically as described^90^. Precultured *E. coli* cells were diluted to 0.01 *A*_600_, and then 2 μL diluted cells was inoculated at the center of the soft agar plate (1% tryptone, 1% NaCl, and 0.3% agar). The plates were cultured at 30 °C for 16 h. The swarming diameter of each strain was averaged from five different sets of measurements.

### Mutator assay

The mutator assay was carried out basically as described^52,91,92^. In addition to the Δ*tapT* strain, Δ*mutS*, Δ*miaA*, Δ*yagA* strains were used as the strong mutator, mild mutator, and negative controls, respectively. *E. coli* cells were cultivated at 42 °C for 5 days and then plated on LB plates at every passage as described in the morphological analysis section. At every passage, adequate amounts of the harvested cells were spread on LB agar plates containing 80 μg/mL nalidixic acid and cultured at 37°C overnight. Mutation rate was calculated from the ratio of colony numbers on the LB plates with and without nalidixic acid.

### Northern blotting

Total RNAs (4 μg) from WT HEK293T and *DTWD1*/*DTWD2* double-KO cells were dissolved by 10% denaturing PAGE, stained with SYBR safe (Invitrogen), and blotted onto a nylon membrane (Amersham Hybond N+; GE Healthcare) in 0.5 × TBE using a Transblot Turbo apparatus (Bio-Rad). Hybridization was performed essentially according to the manufacturer’s instructions (PerfectHyb; TOYOBO) at 42 °C with 2 pmol of 5′-^32^P-labeled oligonucleotides (Supplementary Table 1) complementary to each target tRNA. The membrane was washed three times with 2 × SSC, dried, and exposed to an imaging plate (BAS-MS2040; Fujifilm). Radioactivity was visualized using an FLA-7000 imaging analyzer (Fujifilm).

### Immunostaining

Hela cells (4.0 × 10^5^ cells per dish) were transfected with the pcDNA-3.1 vectors (Invitrogen) bearing the open reading frame of *DTWD1*, *DTWD2L,* or *DTWD2S*, each of which expresses the C-terminally flag-tagged protein. The cells were fixed with 3.7% formaldehyde, permeabilized with 1% Triton X-100, and blocked with 20% Ezblockchemi (ATTO). Immunostaining was performed with anti-DYKDDDDK IgG antibody (1:1000) (014–22383, Wako) for the primary antibody and anti-mouse IgG Alexa fluor 488 (1:1000) (A-11001, Invitrogen) for the secondary antibody. Then, cells were stained with DAPI (1:10,000). The pictures were taken using DMI 6000 (Leica).

### Reporting Summary

Further information on research design is available in the Nature Research Reporting Summary linked to this Article.

## Supplementary Information

Supplementary Information

Peer Review File

Reporting Summary

## Data Availability

A reporting summary for this article is available as a Supplementary Information file. The source data underlying Figs. 2f, 3b–d, and 5a and Supplementary Figs. 3b, 4a, b, and 7a, b are provided as a Source Data file. All data supporting the findings in this study are available from the corresponding author upon reasonable request.

## Source Data

Source Data
