# Supplementary material for: Biogenesis and functions of aminocarboxypropyluridine in tRNA
Source: Nat Commun. 2019 Dec 5;10:5542. doi: 10.1038/s41467-019-13525-3 (PMC6895100; doi:10.1038/s41467-019-13525-3)
Supplement: Supplementary file 1 — Supplementary Information [file 41467_2019_13525_MOESM1_ESM.pdf]

Supplementary information

Biogenesis and functions of aminocarboxypropyluridine in tRNA

Takakura et al.

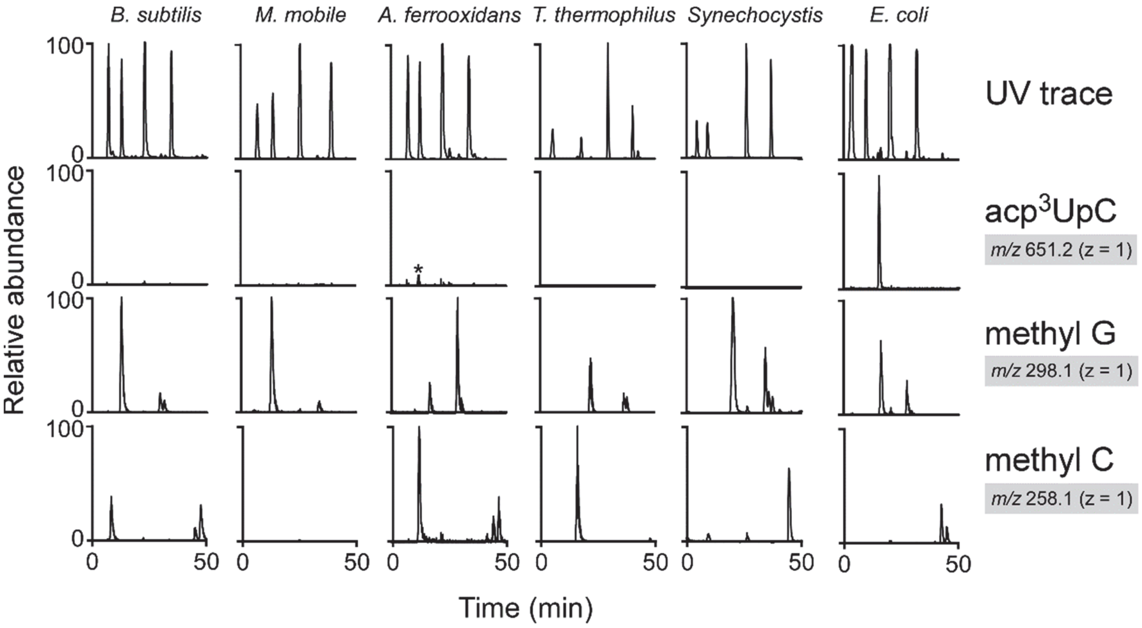

Supplementary Figure 1. Mass spectrometric nucleoside analyses of tRNA modifications in various bacteria.

Top panels represent UV trace. Second, third and fourth panels show XICs of the proton adducts of acp^{3}UpC, methylguanosines and methylcytidines, respectively. Asterisk indicates nonspecific peak with the same *m/z* value.

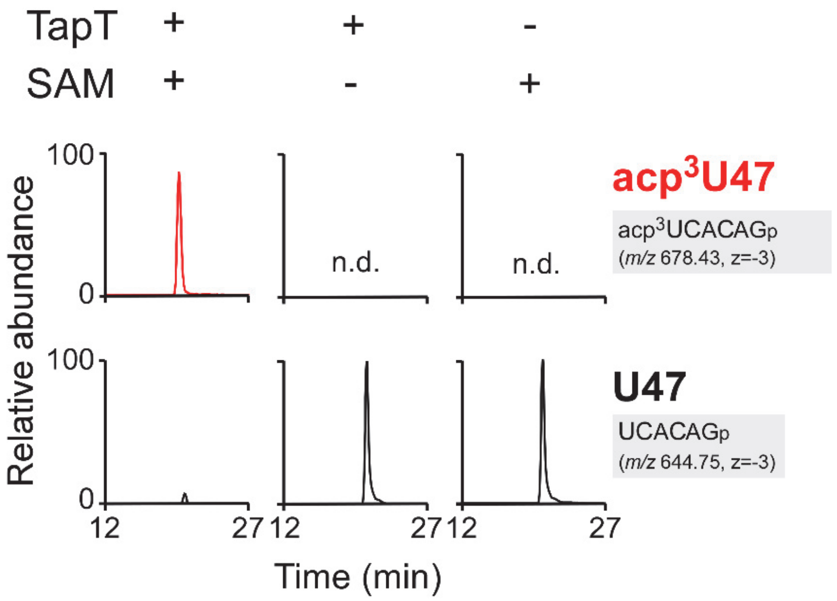

Supplementary Figure 2. *In vitro* reconstitution of acp^{3}U47 in tRNA^{Met} transcript by TapT.

In vitro acp^{3}U formation was performed in the presence or absence of TapT and SAM. XICs show negative ions of the RNase T_1-digested fragments with (upper panels) or without (lower panels) acp^{3}U47.

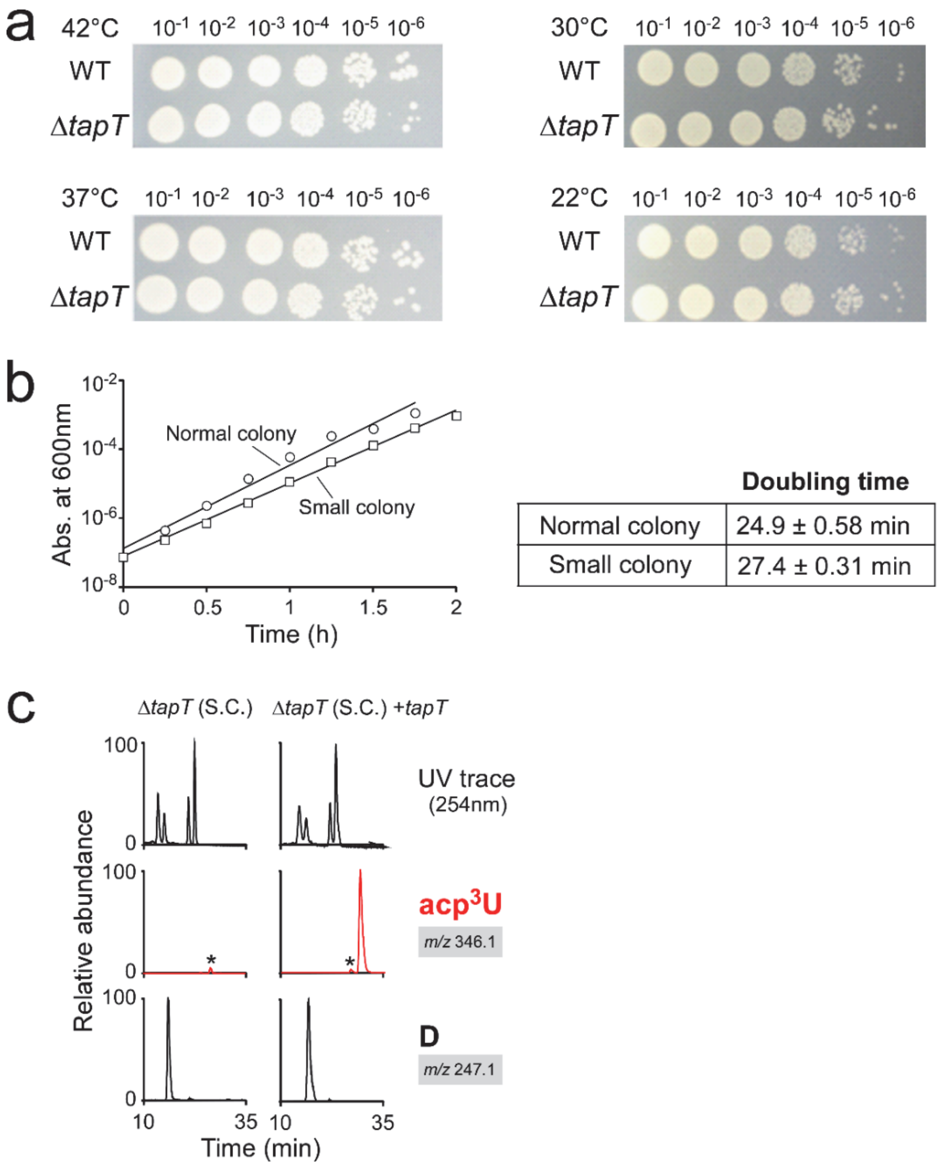

Supplementary Figure 3. Growth and morphological properties of Δ*tapT* strain.

(a) Overnight full-growth cultures were serially diluted (A_{600}, 10^{-1} to 10^{-6}), spotted onto LB plates, and cultivated at 42°C, 37°C, 30°C, or 22°C.

(b) Growth curves of small colony-derived cells (square) and the normal colony-derived cells (circle) cultured at 37°C in LB medium. The doubling time of each strain represents average value of biological triplicates, with s.d. Source data are provided as a Source Data file.

(c) Mass spectrometric nucleoside analyses of tRNA modifications from Δ*tapT* (left panels), and Δ*tapT* rescued by plasmid-encoded *tapT* (right panels). Top panels are UV trace at 254 nm. XICs show the proton adducts of nucleosides for acp^{3}U (middle) and D (bottom). S.C. stands for small colony. Asterisks indicate nonspecific peaks with the same *m/z* value.

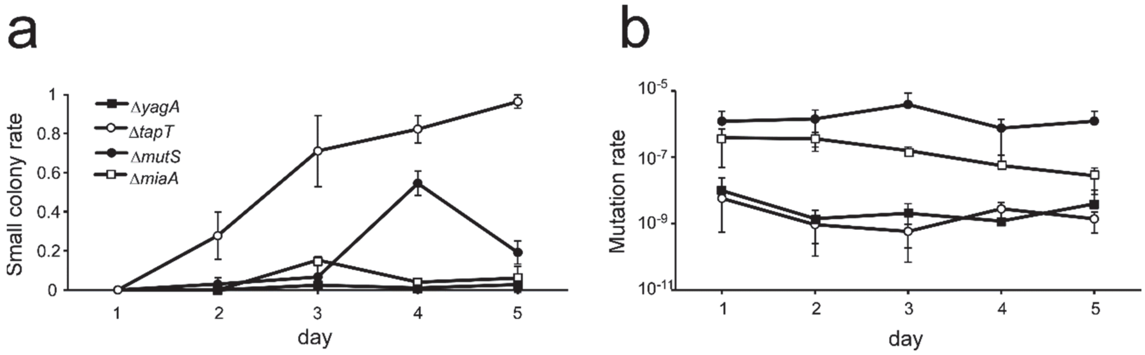

Supplementary Figure 4. Mutation rate does not increase in Δ*tapT* strain. Frequencies of small colony appearance (a) and mutation ratio (b) in Δ*yagA* (closed square), Δ*tapT* (open circle), Δ*mutS* (closed circle), and Δ*miaA* (open square) cultivated continuously at 42°C. Data represent average values of biological triplicates, with s.d. Source data are provided as a Source Data file.

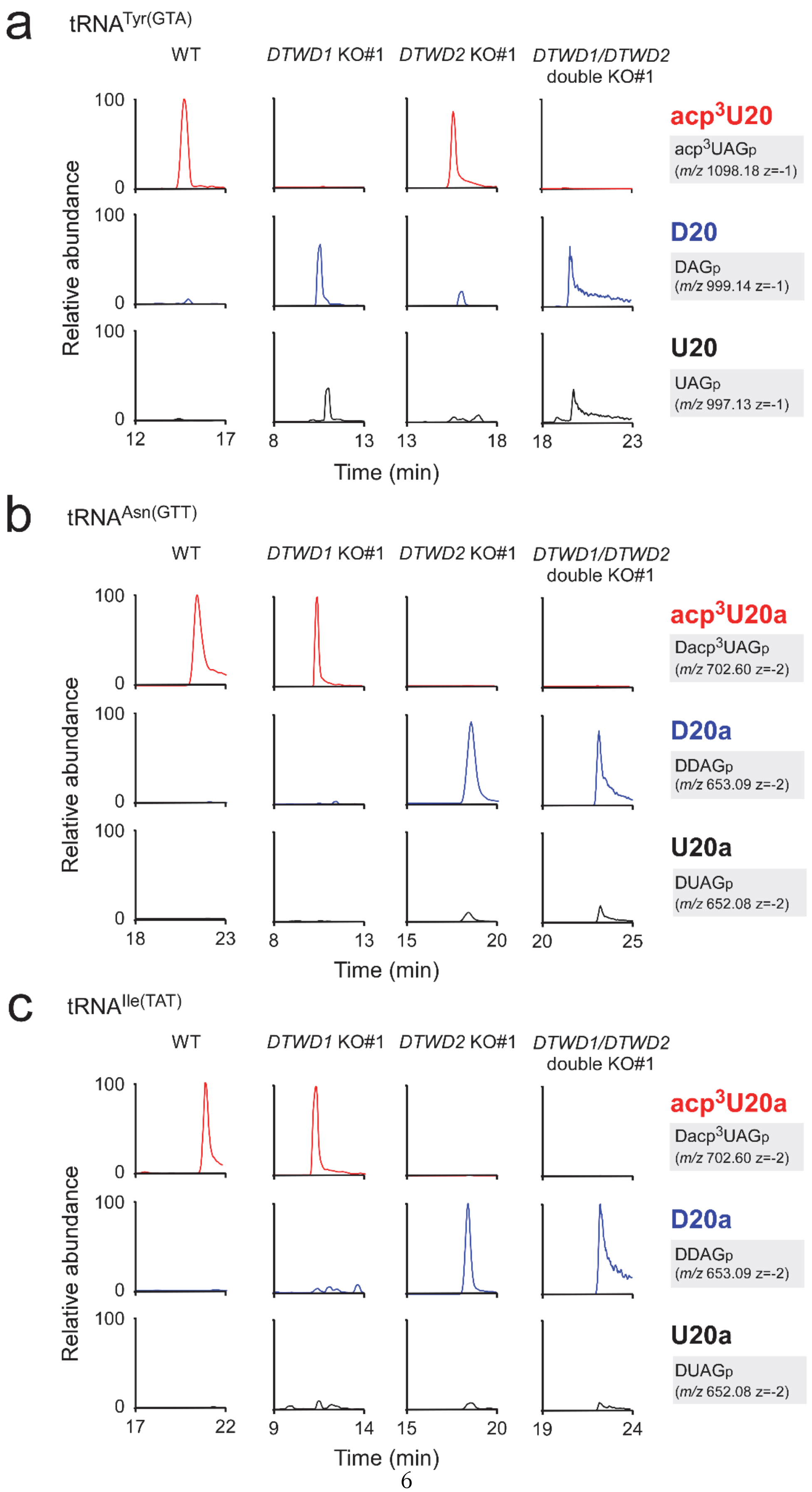
a
tRNA Tyr(GTA)
WT
DTWD1 KO#1
DTWD2 KO#1
DTWD1/DTWD2 double KO#1
Relative abundance
100
0
acp3U20
acp3UAGp (m/z 1098.18 z=-1)
D20
DAGp (m/z 999.14 z=-1)
U20
UAGp (m/z 997.13 z=-1)
12 17
8 13
13 18
18 23
Time (min)
b
tRNA Asn(GTT)
WT
DTWD1 KO#1
DTWD2 KO#1
DTWD1/DTWD2 double KO#1
Relative abundance
100
0
acp3U20a
Dacp3UAGp (m/z 702.60 z=-2)
D20a
DDAGp (m/z 653.09 z=-2)
U20a
DUAGp (m/z 652.08 z=-2)
18 23
8 13
15 20
20 25
Time (min)
c
tRNA Ile(TAT)
WT
DTWD1 KO#1
DTWD2 KO#1
DTWD1/DTWD2 double KO#1
Relative abundance
100
0
acp3U20a
Dacp3UAGp (m/z 702.60 z=-2)
D20a
DDAGp (m/z 653.09 z=-2)
U20a
DUAGp (m/z 652.08 z=-2)
17 22
9 14
15 20
19 24
Time (min)

Supplementary Figure 5. Human *DTWD1* and *DTWD2* are responsible for acp^{3}U formation.

Mass spectrometric analyses of acp^{3}U20 in tRNA$^{Tyr(GTA)}$ (a), and acp^{3}U20a in tRNA$^{Asn(GTT)}$ (b) and tRNA$^{Ile(TAT)}$ (c) isolated from WT (left), *DTWD1* KO (left middle), *DTWD2* KO (right middle), *DTWD1*/*DTWD2* double-KO (right) strains. XICs show corresponding negative ions of the RNase T_1-digested fragments, as indicated on the right-hand side in each chromatogram. Mass spectra of D-containing fragments overlap with the isotopic peaks of U-containing fragments. The peak intensities of these fragments are normalized in consideration of those isotopes.

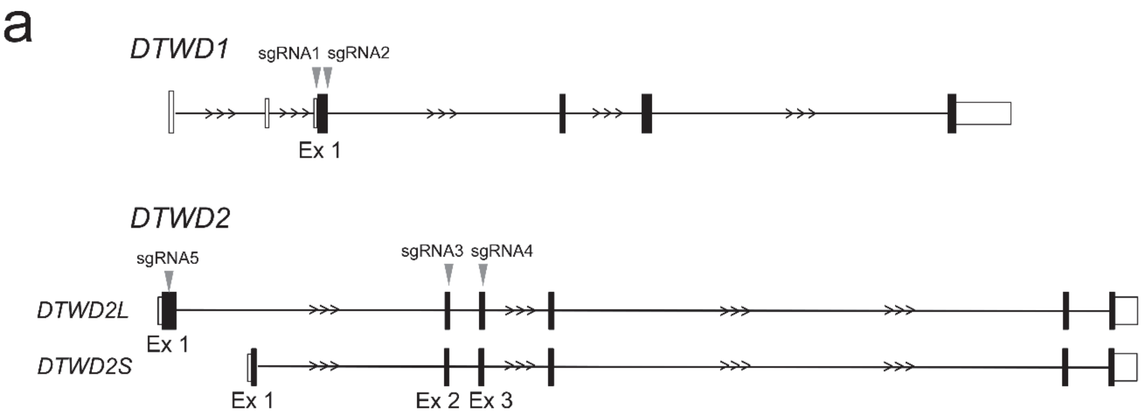

b

sgRNA1	CCTATATTTCTCAACGAAG
sgRNA2	GAATGTTCACTGCTATACA
sgRNA3	GATGCTGAATTATGTACAG
sgRNA4	GGGAGGCATGGCTAGTAG
sgRNA5	GCCCCAGAAGGCGCCAAC

	sgRNA	Genotype
DTWD1 KO#1	sgRNA1	AAT CCA CCT A TA TTT CTC AAA CGA AGT (-5bp)<br>AAT CCA CCT A--(194 bp)--TA TTT CTC AAA CGA AGT
DTWD1 KO#2	sgRNA2	TCC AGA AT G TTC TAC TGC TAT ACA TGT (-10bp)<br>TCC AGA ATTG TTC TAC TGC TAT ACA TGT (+1bp)
DTWD2 KO#1	sgRNA3	CAT ATC TCT ACC CAC TTG TAC ATA ATT CAG CAT CCA (-16bp)
DTWD1DTWD2 double KO#1	sgRNA1<br>sgRNA3	CAT ATC TCT ACC CAC TTG TAC ATA ATT CAG CAT CCA (-5bp)<br>CAT ATC TCT ACC CAC TTG TAC ATA ATT CAG CAT CCA (-1bp)
DTWD1DTWD2 double KO#2	sgRNA2<br>sgRNA4	GTT CCT CTA CTA GCA GCA TGC CTC CCC CAG (-1bp)<br>GTT CCT CTA CTA GCA GCA TGC CTC CCC CAG (-4bp)
DTWD2L KO#1	sgRNA5	TGA GGC CCC AGA AGG CCG CGC AAC GGG CTC (-22bp)<br>TGA GGC CCC AGA AGG CCG CGC AAC GGG CTC (-11bp)

Supplementary Figure 6. Construction of human *DTWD1* and *DTWD2* knockout strains.

(a) Diagram of the human *DTWD1* and *DTWD2* genes, with the sites targeted by sgRNAs.

(b) Sequences of sgRNAs that target human *DTWD1* and *DTWD2* genes (left panel). Genotypes of human *DTWD1* and *DTWD2* KO strains (right panel). The indels (insertions and deletions) in each strain are shown in red letters.

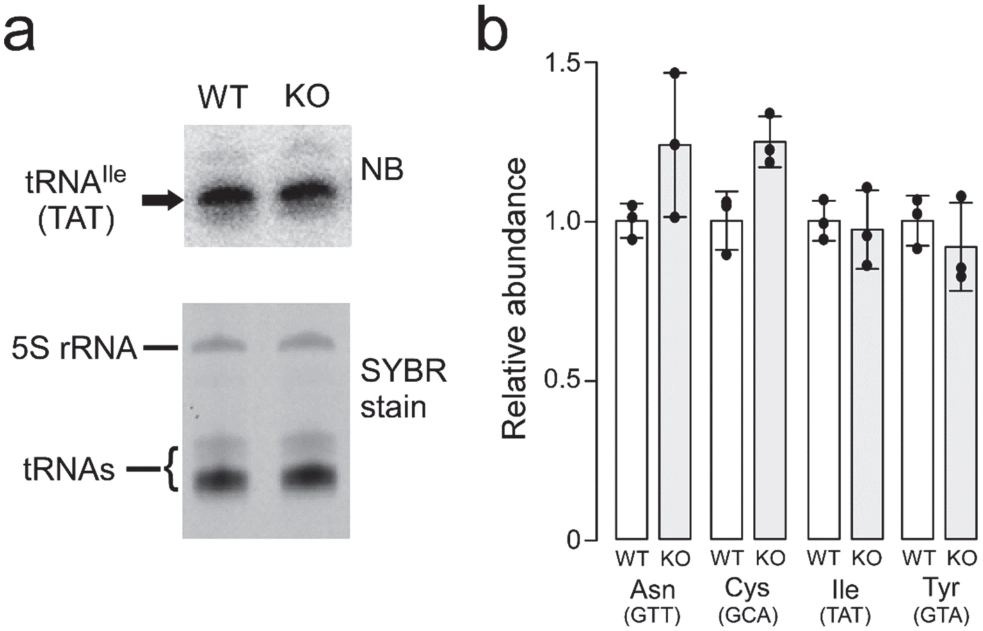

Supplementary Figure 7. Steady-state levels of cytoplasmic tRNAs in the *DTWD1*/*DTWD2* double-KO strain.

(a) Northern blotting (upper panel) of cytoplasmic $tRNA^{Ile(TAT)}$ in total RNAs of WT HEK293T and the double-KO strain. The lower panel shows the gel stained by SYBR Gold. Source data are provided as a Source Data file.

(b) Relative abundance of steady-state levels of tRNAs bearing acp^3U modification. The band intensity of the respective tRNA in the Northern blotting was normalized against the intensity of 5S rRNA stained in the gel. Data represent average values of biological triplicates, with s.d. Source data are provided as a Source Data file.

a

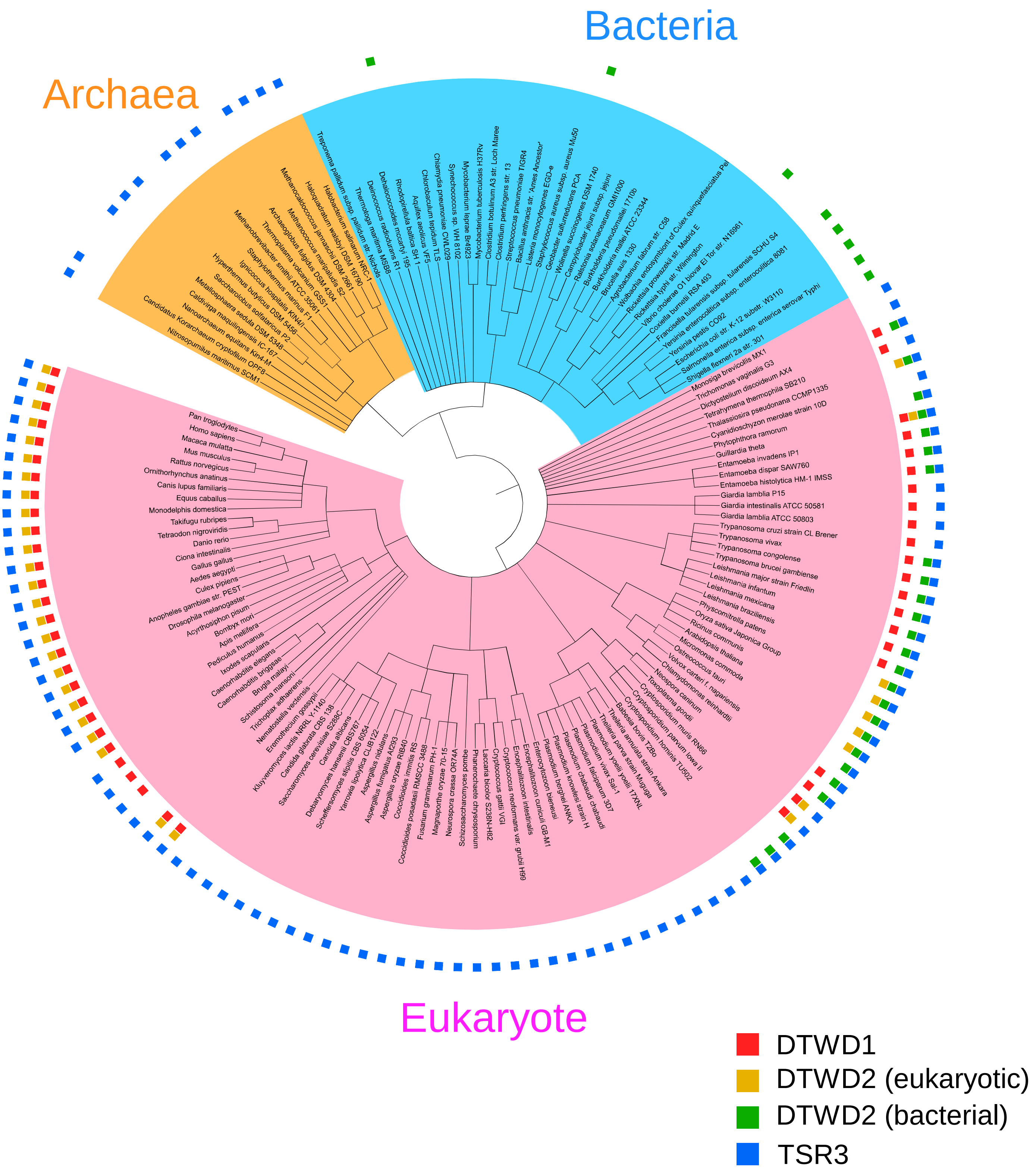

Bacteria
Archaea
Eukaryote
DTWD1
DTWD2 (eukaryotic)
DTWD2 (bacterial)
TSR3

b

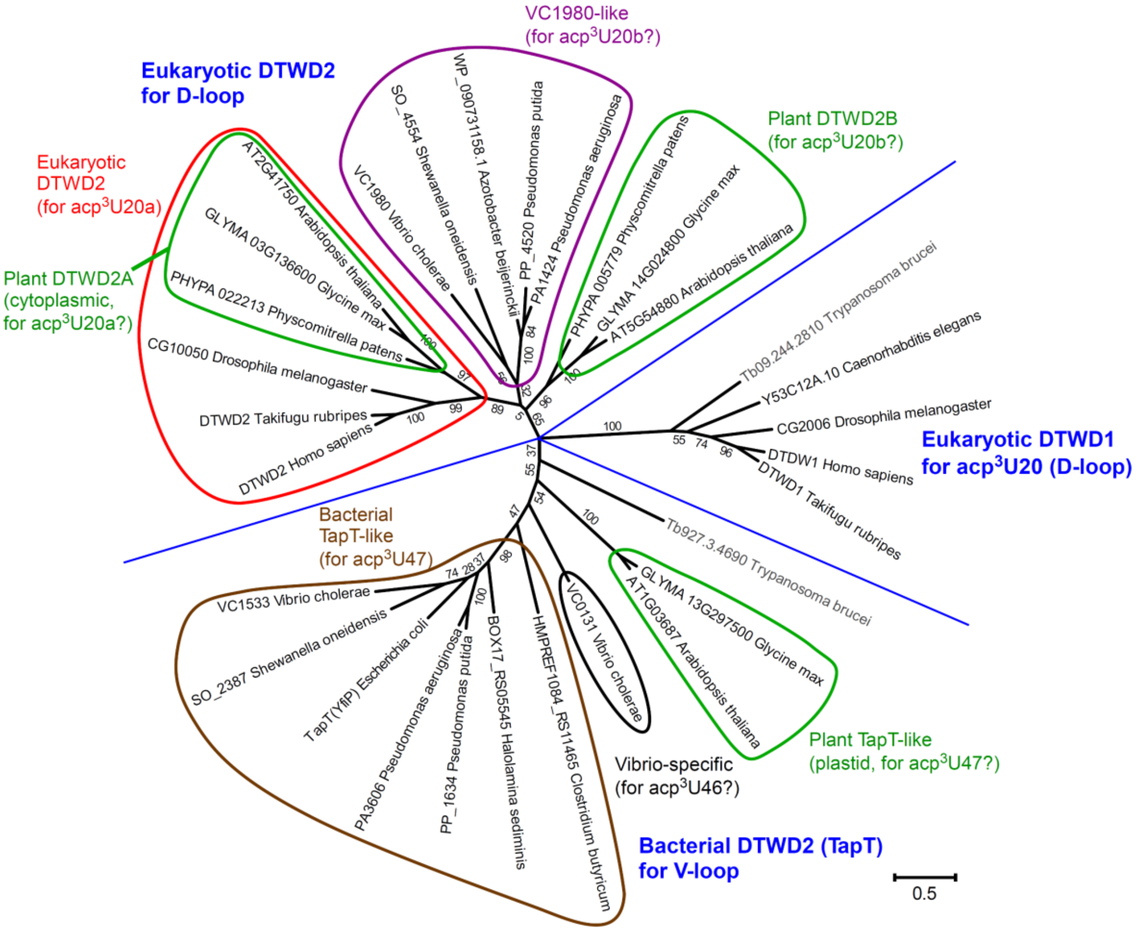

Supplementary Figure 8. Phylogenetic analyses of DTW-containing proteins.

(a) Phylogenetic distribution of TDD superfamily proteins. Organisms and TDD superfamily protein sequences were obtained from OrthoMCL database[1]. A phylogenetic tree of the organisms was built using the common tree tool in NCBI taxonomy database and drawn by the iTOL v4 server[2]. DTWD2 proteins are classified into two subfamilies, eukaryotic and bacterial (TapT), based on the phylogenetic tree of DTWD2 family proteins. The presence of TDD superfamily proteins in each organism was manually checked by NCBI BLAST.

(b) Phylogenetic tree of DTW domain-containing proteins. Multiple alignment of DTW superfamily proteins was constructed using MAFFT. The maximum likelihood tree was constructed using MEGA software[3] with a bootstrap test.

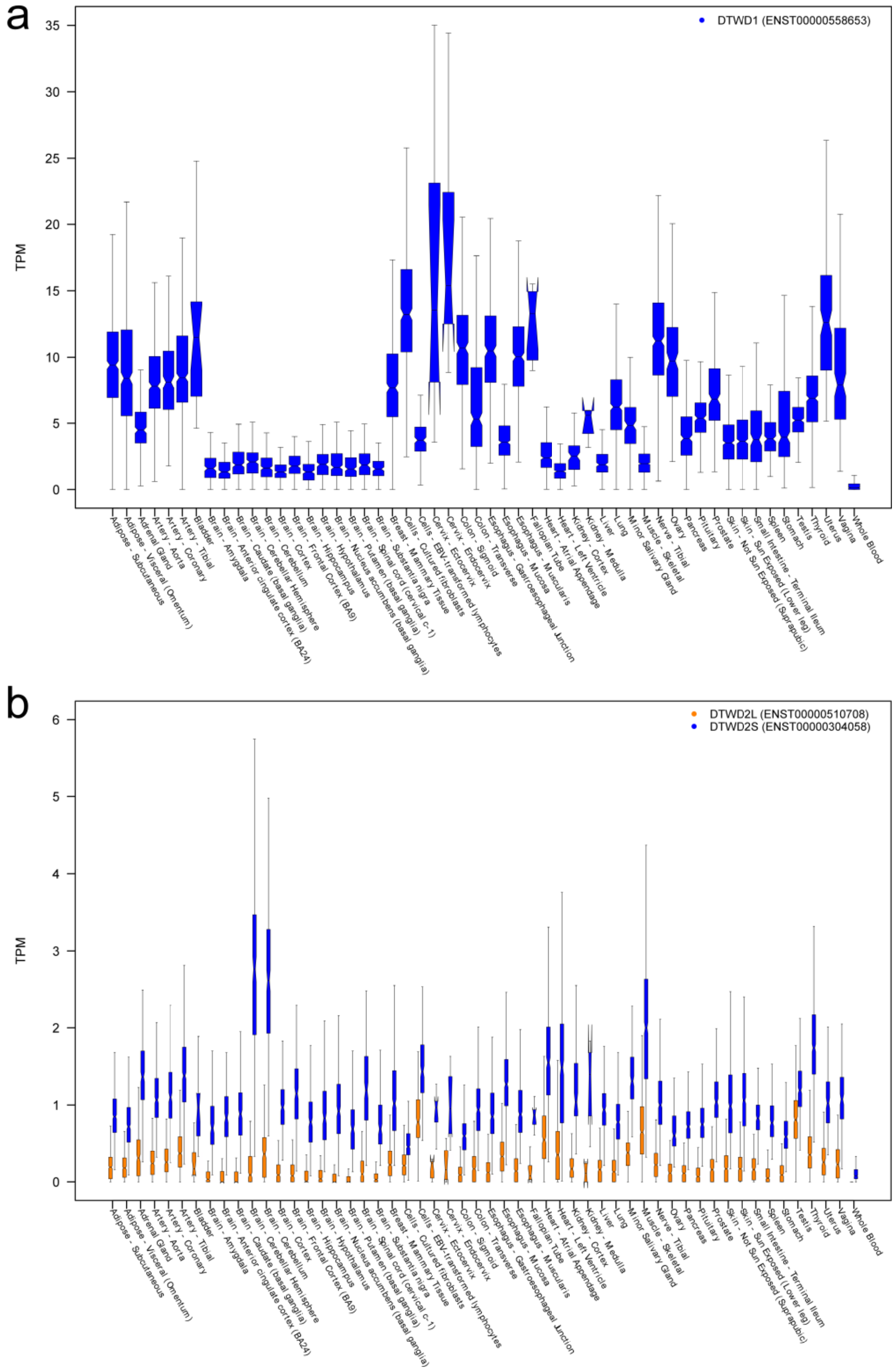

Supplementary Figure 9. Expression profiles of *DTWD1*, *DTWD2L*, and *DTWD2S* in human tissues.

Transcript per million (TPM) of *DTWD1* (a), *DTWD2L*, and *DTWD2S* (b) from various human tissues were obtained from GTEx Analysis V8 [4] and plotted as notched boxplot.

Supplementary References

1 Chen, F., Mackey, A. J., Stoeckert, C. J., Jr. & Roos, D. S. OrthoMCL-DB: querying a comprehensive multi-species collection of ortholog groups. *Nucleic Acids Res* 34, D363-368, doi:10.1093/nar/gkj123 (2006).

2 Letunic, I. & Bork, P. Interactive Tree Of Life (iTOL) v4: recent updates and new developments. *Nucleic Acids Res* 47, W256-W259, doi:10.1093/nar/gkz239 (2019).

3 Kumar, S., Stecher, G., Li, M., Knyaz, C. & Tamura, K. MEGA X: Molecular Evolutionary Genetics Analysis across Computing Platforms. *Molecular biology and evolution* 35, 1547-1549, doi:10.1093/molbev/msy096 (2018).

4 Carithers, L. J. *et al.* A Novel Approach to High-Quality Postmortem Tissue Procurement: The GTEx Project. *Biopreservation and biobanking* 13, 311-319, doi:10.1089/bio.2015.0032 (2015).

Supplementary Table 1. List of primers and probes

Primers

yfiPCDS_Fw	GTGATCTGCATATGACCGAAAACGCTGTTCT
yfiPCDS_Rv	GTGATCTGCTCGAGTTAAACGCTTTCTAACTGTTCTGC
Met_transcript_Fw	GCTAATACGACTCACTATA GGCUACGUAGCUC
Met_transcript_Rv	TGGTGGCTACGACGGGATTCGAACCCG
Met_transcript_body	GGCTACGTAGCTCAGTTGGTTAGAGCACATCACTCATAATGATGGGGTCACAGGTTCGAATC
DTWD1CDS_Fw	GTGATCTGGATATC ATGTCTCTCAATCCACCTATATTTC
DTWD1CDS_Rv	GTGATCTGGCGGCCGC ATGTGTAAGTTTTCCTGTTTCC
DTWD2Lnest_Fw	TTCAATGCCACGGCCTGAC
DTWD2Lnest_Rv	CCTGCTTTTCAGTGAGCCAGTG
DTWD2LCDS_Fw	GTGATCTGGATATC ATGGAGTCGCAGAAAGAG
DTWD2LCDS_Rv	GCGAGCTGGCGGCCGCAATTTTAACACTATTCATTAAC
DTWD2S_Fw	CGGCCTCAGAAAGTGTGTTTG
DTWD2S_Rv	GATATCTGCAGAATTCCACCACAC
sgRNA1_Fw	CACCGCTTCGTTTGAGAAATATAGG
sgRNA1_Rv	AAACCCTATATTTCTCAAACGAAGC
PCR1_Fw	AGTGTGCCAATTTGGTGGTT
PCR1_Rv	TCCCACTTTGCTGAGCTTTT
sgRNA2_Fw	CACCGTGTATAGCAGTAGAACATTC
sgRNA2_Rv	AAACGAATGTTCTACTGCTATACAC
PCR2_Fw	CTTCCATAGCTTCAGAAGATCCCC
PCR2_Rv	CAGAAGTAAATGTGCCCTGCTGAG
sgRNA3_Fw	CACCGATGCTGAATTATGTACAAG
sgRNA3_Rv	AAACCTTGTACATAATTCAGCATC
PCR3_Fw	CTTCGTGTTTCCTCTTGTGGACTC
PCR3_Rv	CCTCTTTGTATAAATGCAGTGGGATTAAGGTC
sgRNA4_Fw	CACCGGGAGGCATGCTGCTAGTAG
sgRNA4_Rv	AAACCTACTAGCAGCATGCCTCCC
PCR4_Fw	TGCCCTAACAGTTAATTTACCTTTCTTCACTGAAGC
PCR4_Rv	CTGTGGTCTTTAGTAAAAGACTTGTGAAAGTTATGC
sgRNA5_Fw	CACCGCCCCAGAAGGCCGCGCAAC
sgRNA5_Rv	AAACGTTGCGCGGCCTTCTGGGGC
PCR5_Fw	TGCACTCAGGCCTCCGCT
PCR5_Rv	TGCGGGCATTGCGCTACG

Probes for RCC and Northern blotting

E. coli Met	TGGTGGCTACGACGGGATTCGAACCTGTGA
human Asn-GTT-2	TGGCGTCCCTGGGTGGGCTCGAACCACCAACCTTTCGGTT
human Cys-GCA-2	TGGAGGGGGCACCCGGATTTGAACCGGGGACCTCTTGATC
human Ile-AAT-1	TGGTGGCCCGTACGGGGATCGAACCCGCGACCTTGGCGTT
human Ile-TAT-2	TGGTGCTCCAGGTGAGGCTCGAACTCACAACCTCGGCATT
human Tyr-GTA-2	TGGTCCTTCGAGCCGGAATCGAACCAGCGACCTAAGGATC
