# Supplementary material for: Biogenesis and functions of aminocarboxypropyluridine in tRNA
Source: Nat Commun. 2019 Dec 5;10:5542. doi: 10.1038/s41467-019-13525-3 (PMC6895100; doi:10.1038/s41467-019-13525-3)
Supplement: Supplementary file 3 — Reporting Summary [file 41467_2019_13525_MOESM3_ESM.pdf]

nature research

Corresponding author(s): Tsutomu Suzuki

Last updated by author(s): Sep 19, 2019

Reporting Summary

Nature Research wishes to improve the reproducibility of the work that we publish. This form provides structure for consistency and transparency in reporting. For further information on Nature Research policies, see Authors & Referees and the Editorial Policy Checklist .

Statistics

For all statistical analyses, confirm that the following items are present in the figure legend, table legend, main text, or Methods section.

n/a	Confirmed	
☐	☒	The exact sample size (n) for each experimental group/condition, given as a discrete number and unit of measurement
☐	☒	A statement on whether measurements were taken from distinct samples or whether the same sample was measured repeatedly
☐	☒	The statistical test(s) used AND whether they are one- or two-sided<br>*Only common tests should be described solely by name; describe more complex techniques in the Methods section.*
☒	☐	A description of all covariates tested
☒	☐	A description of any assumptions or corrections, such as tests of normality and adjustment for multiple comparisons
☐	☒	A full description of the statistical parameters including central tendency (e.g. means) or other basic estimates (e.g. regression coefficient) AND variation (e.g. standard deviation) or associated estimates of uncertainty (e.g. confidence intervals)
☐	☒	For null hypothesis testing, the test statistic (e.g. F, t, r) with confidence intervals, effect sizes, degrees of freedom and P value noted<br>*Give P values as exact values whenever suitable.*
☒	☐	For Bayesian analysis, information on the choice of priors and Markov chain Monte Carlo settings
☒	☐	For hierarchical and complex designs, identification of the appropriate level for tests and full reporting of outcomes
☒	☐	Estimates of effect sizes (e.g. Cohen's d, Pearson's r), indicating how they were calculated

Our web collection on statistics for biologists contains articles on many of the points above.

Software and code

Policy information about availability of computer code

Data collection	Microbial Genome Database(MGBD), NCBI database, Pfam, OrthoMCL database, GTEx Analysis V8 (dbGaP Accession phs000424.v8.p2), Mongo Oligo Mass Calculator v2.08, UV-Vis spectrophotometer (V-630; JASCO), SpectraMax Paradigm MultiMode Detection, FLA-7000 system, LTQ Orbitrap XL, Q Exactive Hybrid Quadrupole-Orbitrap Mass Spectrometer, DMI 6000B
Data analysis	Microsoft Excel(2016), Canvas 16 and ChemDraw Professional 16.0 were used for making figures and data analysis.<br>Qual Browser (in Xcalibur 4.1) was used for mass spectrometric analysis.<br>Muti gauge v3.0 was used for graphical analysis.<br>Basic Local alignment Search Tool (BLAST), MAFFT, iTOL and MEGA were used for phylogenic analyses.

For manuscripts utilizing custom algorithms or software that are central to the research but not yet described in published literature, software must be made available to editors/reviewers. We strongly encourage code deposition in a community repository (e.g. GitHub). See the Nature Research guidelines for submitting code & software for further information.

Data

Policy information about availability of data

All manuscripts must include a data availability statement. This statement should provide the following information, where applicable:

- Accession codes, unique identifiers, or web links for publicly available datasets
- A list of figures that have associated raw data
- A description of any restrictions on data availability

The data supporting the findings are available from the corresponding author upon reasonable request.

Field-specific reporting

Please select the one below that is the best fit for your research. If you are not sure, read the appropriate sections before making your selection.

- [x] Life sciences
- [ ] Behavioural & social sciences
- [ ] Ecological, evolutionary & environmental sciences

For a reference copy of the document with all sections, see nature.com/documents/nr-reporting-summary-flat.pdf

Life sciences study design

All studies must disclose on these points even when the disclosure is negative.

Sample size	Sample size is more than 3 to apply student t-test.
Data exclusions	No data were excluded apart from occational point-outliners (probably due to machine error) during a series of growth rate analysis.
Replication	All attempts at replication were succesful.
Randomization	We did no statistical test that requires randomization of samples.
Blinding	Investigations were not blinded, because blinding would not increase reliability of biochemical and genetic experiments.

Reporting for specific materials, systems and methods

We require information from authors about some types of materials, experimental systems and methods used in many studies. Here, indicate whether each material, system or method listed is relevant to your study. If you are not sure if a list item applies to your research, read the appropriate section before selecting a response.

Materials & experimental systems

n/a	Involved in the study	
[ ]	[x]	Antibodies
[ ]	[x]	Eukaryotic cell lines
[x]	[ ]	Palaeontology
[x]	[ ]	Animals and other organisms
[x]	[ ]	Human research participants
[x]	[ ]	Clinical data

Methods

n/a	Involved in the study	
[x]	[ ]	ChIP-seq
[x]	[ ]	Flow cytometry
[x]	[ ]	MRI-based neuroimaging

Antibodies

Antibodies used	Anti-DYKDDDDK tag, Monoclonal Antibody (Wako, 014-22383, Mouse, lot:CTR5949)<br>Alexa Fluor 488 (Invitrogen, A11001, goat Anti-mouse IgG lot:840881)
Validation	For Anti-DYKDDDDK tag, see this URL below.<br>https://labchem-wako.fujifilm.com/jp/category/01430.html

Eukaryotic cell lines

Policy information about cell lines

Cell line source(s)	HEK293T (CRL-11268) and Hela cells (CCL-2) were used in our research.
Authentication	None of the cell lines were authenticated.
Mycoplasma contamination	All of original cell lines tested showed negative for mycoplasma conatamination.
Commonly misidentified lines<br>(See ICLAC register)	No commonly misidentified lines was used in our research.
