# Supplementary material for: Biogenesis and functions of aminocarboxypropyluridine in tRNA
Source: Nat Commun. 2019 Dec 5;10:5542. doi: 10.1038/s41467-019-13525-3 (PMC6895100; doi:10.1038/s41467-019-13525-3)
Supplement: Supplementary file 2 — Peer Review File [file 41467_2019_13525_MOESM2_ESM.pdf]

REVIEWERS' COMMENTS:

Reviewer #1 (Remarks to the Author):

The manuscript by Takakura et al describes the identification of the acp3U generating enzyme in bacteria and eukaryotes, which had been missing from the list of known tRNA modification enzymes for many years. The experiments described here beautifully and convincingly demonstrate that the gene called by the authors TapT, and its human homologs DTWD1 and DTWD2 are the missing enzymes responsible for this activity. State of the art techniques are applied to identify and map the acp3 modification, and the genetic analysis provides incontrovertible evidence for the newly-identified functions. While there is no satisfying molecular explanation for the small colony phenotype that is exhibited by bacterial tapT deletion strains at high temperature, the authors exhaust some of the most likely possible reasons for this. In this case, additional studies to address the source of this phenotype would seem to be beyond the scope of the current work. Likewise, the molecular basis for the growth phenotype exhibited in the double KO strain in human cells remains unknown, but the northern experiments clearly rule out a possible effect on steady-state tRNA levels, and this result on its own is valuable. In short, this represents a very nice contribution to the field of RNA modification.

I have a minor criticism about the conclusion that acp3U20a formation occurs both in cytoplasm and nucleus, since it has not been demonstrated by individual knockout that both isoforms of DTWD2 are functional (CRISPR KO strains target both isoforms). It is certainly possible that only one is the relevant catalytic isoform and in the absence of individual KO strains targeting specific isoforms, this conclusion is not supported by the data.

Given the broad evolutionary impact of the work, the figure S8 that is referred to in the discussion would be better presented in the main text (rather than supplemental). Also, it would be helpful to highlight the 2 T. brucei enzymes on the phylogeny so that this can be appreciated better.

Minor editorial issues

1) figure 1b citation on p 4 referring to Tsr3 structure is not entirely appropriate (since modified pseudoU and U is shown), better to remove this one and leave citation of the figure on next page
2) labels on Figure 2f should be corrected to standardize significant figures (and match numbers in text)
3) Figure S6a- the gene diagram of DTWD2 is somewhat confusing to follow since it is shown in opposite orientation (presumably because of the coding orientation relative to DTWD1 in the genome). However, this is not really relevant for the understanding of the gene, and it would be easier for the reader if it were switched to an orientation where the exons are in order from left to right (like DTWD1).
4) Final sentence in the first paragraph on p. 6 is confusing as written- doesn't quite get across the point about the TDD superfamily (named and defined by the 3 enzymes Tsr3, DTWD1, DTWD2).
5) Minor English language editing is needed throughout the manuscript

Reviewer #2 (Remarks to the Author):

This manuscript describes the elegant discovery and characterization of a key step in the biosynthetic pathway of the widely conserved tRNA modification, acp3U. The authors used their trademark clever coupling of comparative genomics with L-MS nucleoside analysis to discover that the yfiP gene

encodes the tRNA aminocarboxypropyl transferase (TapT) responsible for acp3U formation in E. coli. The in vitro reconstitution of acp3U47 biosynthesis coupled with LC-MS structural analysis rigorously confirmed the biochemical assignment. The authors then cleverly correlated tRNA sequencing mutations with the presence of acp3U in human tRNAs and subsequently confirmed the modification and its location by LC-MS. Again, comparative genomics coupled with LC-MS in knock-down strains led to the assignment of the DTWD1 and DTWD2 genes as the human homologs of the tapT gene in E. coli. Altogether, this well-executed and well-controlled study elegantly revealed a novel biosynthetic pathway for a poorly understood but widely conserved tRNA modification, with the biosynthetic genes linked to a variety of human diseases. This work would be of interest to readers of Nature Communications.

Minor problems:

• The introduction is rambling and a bit unfocused, with a general review of many types and locations of tRNA modifications. The authors have a great story to tell about acp3U so the introduction could be more focused.

• Figure 2e: The CID spectrum needs to show the m/z values at the major signals, including the assigned y-ions.

Response to the comments by the reviewers

We appreciate the reviewers for spending your precious time to review our manuscript, and giving us many positive comments and appropriate advices to improve it.

Response to the Reviewer #1's comments

The manuscript by Takakura et al describes the identification of the acp3U generating enzyme in bacteria and eukaryotes, which had been missing from the list of known tRNA modification enzymes for many years. The experiments described here beautifully and convincingly demonstrate that the gene called by the authors TapT, and its human homologs DTWD1 and DTWD2 are the missing enzymes responsible for this activity. State of the art techniques are applied to identify and map the acp3 modification, and the genetic analysis provides incontrovertible evidence for the newly-identified functions. While there is no satisfying molecular explanation for the small colony phenotype that is exhibited by bacterial tapT deletion strains at high temperature, the authors exhaust some of the most likely possible reasons for this. In this case, additional studies to address the source of this phenotype would seem to be beyond the scope of the current work. Likewise, the molecular basis for the growth phenotype exhibited in the double KO strain in human cells remains unknown, but the northern experiments clearly rule out a possible effect on steady-state tRNA levels, and this result on its own is valuable. In short, this represents a very nice contribution to the field of RNA modification.

We appreciate deep understanding our findings and many positive comments. Regarding the small colony phenotype and growth reduction in the double KO strain, we will tackle to reveal molecular mechanisms for these issues in our future papers.

I have a minor criticism about the conclusion that acp3U20a formation occurs both in cytoplasm and nucleus, since it has not been demonstrated by individual knockout that both isoforms of DTWD2 are functional (CRISPR KO strains target both isoforms). It is certainly possible that only one is the relevant catalytic isoform and in the absence of individual KO strains targeting specific isoforms, this conclusion is not supported by the data.

Thank you very much for pointing out this critical issue. Two isoforms of human

DTWD2, DTWD2L and DTWD2S, with different first exons are produced by alternative splicing. To check whether both isoforms actually have activity, we have constructed *DTWD2L*-specific KO cell line by targeting the exon 1 of *DTWD2L*. We isolated $tRNA^{Ile(AAT)}$ from *DTWD2L* KO cells, and analyzed the status of acp^3U20a modification. As shown in new Figure 4b, both acp^3U20a and acp^3D20a partially decreased, and instead D20a increased. This data clearly demonstrated that DTWD2L actually has an activity for $acp^3U(D)20a$ formation, and DTWD2S is redundantly responsible for the remaining modification. The only difference of these two isoforms is their N-terminal sequences that are far from the catalytic site of DTW domain. So, the enzymatic activity found in both isoforms is reasonable conclusion.

Given the broad evolutionary impact of the work, the figure S8 that is referred to in the discussion would be better presented in the main text (rather than supplemental). Also, it would be helpful to highlight the 2 T. brucei enzymes on the phylogeny so that this can be appreciated better.

Thank you very much for your suggestion. We considered this suggestion seriously and discussed with the coauthors and the editor. We'd like to show this figure in supplementary information, because this is a phylogenetic distribution of TDD superfamily, a part of which was previously analyzed and reported by the other group. In addition, it is very complicated figure. So, it is shown as Supplementary Figure 8. Please notice that *T. brucei* enzymes are highlighted with different color.

Minor editorial issues

1) figure 1b citation on p 4 referring to Tsr3 structure is not entirely appropriate (since modified pseudoU and U is shown), better to remove this one and leave citation of the figure on next page

We appreciate pointing out this, and removed Figure 1b citation from here.

2) labels on Figure 2f should be corrected to standardize significant figures (and match numbers in text)

As suggested, the Tm values are unified to 3 significant figures with 2 significant figures for their standard deviations.

3) Figure S6a- the gene diagram of DTWD2 is somewhat confusing to follow since it is shown in opposite orientation (presumably because of the coding orientation relative to DTWD1 in the genome). However, this is not really relevant for the understanding of the gene, and it would be easier for the reader if it were switched to an orientation where the exons are in order from left to right (like DTWD1).

As suggested, the gene orientation of DTWD2 in Figure S6a has been switched from left to right.

4) Final sentence in the first paragraph on p. 6 is confusing as written- doesn't quite get across the point about the TDD superfamily (named and defined by the 3 enzymes Tsr3, DTWD1, DTWD2).

We rephrased this sentence by defining the TDD superfamily which includes the TSR3 (COG2042), DTWD1 (KOG3795) and DTWD2 (KOG4382) families.

5) Minor English language editing is needed throughout the manuscript

The revised version has been checked carefully and English grammar and usage were edited appropriately.

Response to the Reviewer #2's comments

This manuscript describes the elegant discovery and characterization of a key step in the biosynthetic pathway of the widely conserved tRNA modification, acp3U. The authors used their trademark clever coupling of comparative genomics with L-MS nucleoside analysis to discover that the yfiP gene encodes the tRNA aminocarboxypropyl transferase (TapT) responsible for acp3U formation in E. coli. The in vitro reconstitution of acp3U47 biosynthesis coupled with LC-MS structural analysis rigorously confirmed the biochemical assignment. The authors then cleverly correlated tRNA sequencing mutations with the presence of acp3U in human tRNAs and subsequently confirmed the modification and its location by LC-MS. Again, comparative genomics coupled with LC-MS in knock-down strains led to the assignment of the DTWD1 and DTWD2 genes as the human homologs of the tapT gene in E. coli. Altogether, this well-executed and well-controlled study elegantly revealed a

novel biosynthetic pathway for a poorly understood but widely conserved tRNA modification, with the biosynthetic genes linked to a variety of human diseases. This work would be of interest to readers of Nature Communications.

We really appreciate positive estimation of our findings.

Minor problems:

• The introduction is rambling and a bit unfocused, with a general review of many types and locations of tRNA modifications. The authors have a great story to tell about acp3U so the introduction could be more focused.

As suggested, we revised the introduction to focus acp^{3}U modification.

• Figure 2e: The CID spectrum needs to show the m/z values at the major signals, including the assigned y-ions.

As suggested, the major product ions in the CID spectrum are labeled with *m/z* values.
